# Mitochondria Matter: Systemic Aspects of Nonalcoholic Fatty Liver Disease (NAFLD) and Diagnostic Assessment of Liver Function by Stable Isotope Dynamic Breath Tests

**DOI:** 10.3390/ijms22147702

**Published:** 2021-07-19

**Authors:** Agostino Di Ciaula, Giuseppe Calamita, Harshitha Shanmugam, Mohamad Khalil, Leonilde Bonfrate, David Q.-H. Wang, Gyorgy Baffy, Piero Portincasa

**Affiliations:** 1 Clinica Medica “A. Murri”, Department of Biomedical Sciences & Human Oncology, University of Bari Medical School, 70124 Bari, Italy; agostinodiciaula@tiscali.it (A.D.C.); harshithashanmugam993@gmail.com (H.S.); mak_37_47@hotmail.com (M.K.); leonilde.bonfrate@uniba.it (L.B.); 2 Department of Biosciences, Biotechnologies and Biopharmaceutics, University of Bari “Aldo Moro”, 70100 Bari, Italy; giuseppe.calamita@uniba.it; 3 Marion Bessin Liver Research Center, Einstein-Mount Sinai Diabetes Research Center, Department of Medicine and Genetics, Division of Gastroenterology and Liver Diseases, Albert Einstein College of Medicine, Bronx, NY 10461, USA; david.wang@einsteinmed.org; 4 Department of Medicine, VA Boston Healthcare System and Brigham and Women’s Hospital, Harvard Medical School, Boston, MA 02130, USA; GBaffy@bwh.harvard.edu

**Keywords:** breath test, hepatic mitochondrial function, hepatocellular carcinoma, ketoisocaproic acid, liver diseases, liver steatosis, methionine, methacetin, octanoic acid, β-oxidation

## Abstract

The liver plays a key role in systemic metabolic processes, which include detoxification, synthesis, storage, and export of carbohydrates, lipids, and proteins. The raising trends of obesity and metabolic disorders worldwide is often associated with the nonalcoholic fatty liver disease (NAFLD), which has become the most frequent type of chronic liver disorder with risk of progression to cirrhosis and hepatocellular carcinoma. Liver mitochondria play a key role in degrading the pathways of carbohydrates, proteins, lipids, and xenobiotics, and to provide energy for the body cells. The morphological and functional integrity of mitochondria guarantee the proper functioning of β-oxidation of free fatty acids and of the tricarboxylic acid cycle. Evaluation of the liver in clinical medicine needs to be accurate in NAFLD patients and includes history, physical exam, imaging, and laboratory assays. Evaluation of mitochondrial function in chronic liver disease and NAFLD is now possible by novel diagnostic tools. “Dynamic” liver function tests include the breath test (BT) based on the use of substrates marked with the non-radioactive, naturally occurring stable isotope ^13^C. Hepatocellular metabolization of the substrate will generate ^13^CO_2_, which is excreted in breath and measured by mass spectrometry or infrared spectroscopy. Breath levels of ^13^CO_2_ are biomarkers of specific metabolic processes occurring in the hepatocyte cytosol, microsomes, and mitochondria. ^13^C-BTs explore distinct chronic liver diseases including simple liver steatosis, non-alcoholic steatohepatitis, liver fibrosis, cirrhosis, hepatocellular carcinoma, drug, and alcohol effects. In NAFLD, ^13^C-BT use substrates such as α-ketoisocaproic acid, methionine, and octanoic acid to assess mitochondrial oxidation capacity which can be impaired at an early stage of disease. ^13^C-BTs represent an indirect, cost-effective, and easy method to evaluate dynamic liver function. Further applications are expected in clinical medicine. In this review, we discuss the involvement of liver mitochondria in the progression of NAFLD, together with the role of ^13^C-BT in assessing mitochondrial function and its potential use in the prevention and management of NAFLD.

## 1. Introduction

The liver plays a key role in lipid homeostasis, with steps including the synthesis, oxidation, and transport of free fatty acids (FFA), triglycerides (TG), cholesterol, and bile acids (BA). Chronic liver diseases encompass a spectrum of conditions ranging from metabolic to viral, alcohol-related diseases, drug-related diseases, autoimmune diseases, and tumours. The hepatocyte can be damaged by various hits and intracellular organelles can be part of the dysfunctional cell, with changes including microsomal hypertrophy, mitochondrial damage by free fatty acids overload and insufficient β-oxidation and activation of peroxisomal metabolism.

Growing evidence points to dysfunctional mitochondria as key contributors in the pathogenesis of the chronic metabolic conditions (i.e., obesity, metabolic syndrome and type 2 diabetes mellitus) frequently linked to liver disease. These processes act through pathways leading to oxidative stress, chronic inflammation, and insulin resistance. Thus, diagnostic techniques able to early detect and monitor mitochondrial dysfunction have great relevance in terms of possible primary/secondary prevention measures and of therapies specifically targeting liver mitochondria [1,2,3].

In this paper we will focus on nonalcoholic fatty liver disease (NAFLD) with emphasis on mitochondrial dysfunction and the role of novel “dynamic” noninvasive breath test (BT) to assess mitochondrial function. We will also point to current potential therapeutic approaches targeting mitochondria in NAFLD.

## 2. Mitochondrial Function in the Liver

### 2.1. General Features of Mitochondria

Mitochondria are intracellular organelles that provide energy for the body cells. In the liver there are about 500–4000 mitochondria per cell [4] equalling about 18% of the entire cell volume [5]. Mitochondria play a key role in the metabolic pathways and signalling networks [6]. They participate in degrading pathways of carbohydrates, proteins, lipids, and xenobiotics [7,8], and ultimately generate ATP as energetic source [6,7,8]. The morphological and functional integrity of mitochondria maintain functioning networks and pathways inside the mitochondria and in the cell. The fat and energy balance in hepatocytes is regulated by mitochondrial activities, including FFA β-oxidation, electron transfer and production of ATP and ROS [9]. Essential elements include mitochondrial DNA (mtDNA), membrane constituents, lipoprotein trafficking, pro- and anti-oxidant balance, and metabolic demand and supply [10].

### 2.2. The Fate of Free Fatty Acids 

The routes providing the circulating (long-chain) FFA to the liver and their mitochondrial fate is of relevance for ATP production and hepatocyte health maintenance. About 60% of circulating FFA derive from lipolysis of TG in adipose tissue [11], and enter the hepatocyte by using the transporters FFA translocase/CD36, fatty acid binding protein (FABP), and caveolin-1. About 15% FFA are of dietary origin and contained in TG within ApoE-enriched chylomicrons. Chylomicrons are assembled in the enterocyte following dietary fat digestion in the intestinal lumen. This step occurs during emulsion and micellization of fat by the bile acid (BA) pool made of both primary and secondary tauro-, glycol-conjugated BA [12,13]. In the hepatocyte chylomicron remnants bind specific membrane receptors taht have a high affinity for the surface protein ApoE. Lastly, about 35% of FFA in the hepatocyte originate from de novo lipogenesis (DNL) from dietary carbohydrates (glucose converted to pyruvate during glycolysis). The FFA pool in the hepatocyte provides the substrate for re-esterification with glycerol to form TG via the key enzymes, diglyceride acyltransferase (DGAT)1 and DGAT2. This amount of TG serves as stored fat as lipid droplets in small amounts, i.e., less than 5% of cell content. When needed, TG can be hydrolysed by hydrolases, e.g., the patatin-like phospholipase domain-containing protein 3-PNPLA3 (adiponutrin)—to release FFA in the cytosol [14,15]. The TG pool also provides FFA for two major routes of elimination [16]. (a) export to blood as TG within very-low density lipoproteins (VLDL) assembled in the endoplasmic reticulum [17]; (b) β-oxidation of FFA in mitochondria. In the Golgi apparatus the apolipoprotein B (ApoB) undergoes disulphide bond formation and association with TG (by protein disulphide isomerase and microsome triglyceride transfer protein (MTP)) [18]. Of note, increased intake of sucrose in the mice model leads to rapid development of hyperinsulinemia, hepatosteatosis, and insulin resistance. Furthermore, insulin enhances hepatic expression of the FA transporter CD36 involving a PPAR-γ-dependent mechanism. In the general scenario, these results indicate that hyperinsulinemia is an early and potent inducer of hepatosteatosis, insulin resistance, and dysglycaemia. A further step is the progression to type 2 diabetes and NAFLD. In addition, during conditions of hyperinsulinemia, dysfunctional insulin clearance becomes evident, due to abnormal insulin degrading enzyme regulation. This step, in turn, directly impairs postprandial hepatic glucose disposal and increases susceptibility to dysmetabolic conditions, including fatty liver, mitochondrial dysfunction, especially in the setting of Western diet/lifestyle.

### 2.3. β-Oxidation of FFA in Mitochondria

**Figure 1:**
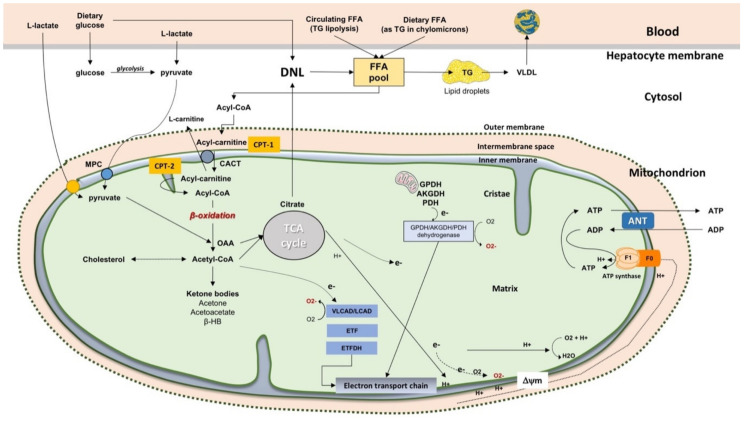
Mitochondrial function in the liver. Oxidative metabolism and hepatocyte energy homeostasis depend on FFA β-oxidation, the tricarboxylic acid cycle (TCA), electron flow along the electron transport chain, electrochemical proton gradient generation, and ATP synthesis. Ketone bodies are produced due to an absence of oxaloacetate used in gluconeogenesis (e.g., starvation and diabetes). Starting from blood, dietary glucose, dietary FFA (as TG within chylomicrons) and FFA circulating after TG lipolysis, enrich the FFA pool in the hepatocyte. During fatty acid synthesis, glucose from dietary sources during glycolysis is converted to pyruvate which can enter the mitochondrion via the mitochondrial pyruvate carrier (MPC). Pyruvate also can be synthesised from L-lactate after transport of L-lactate in the matrix, via its own carrier, and oxidation via the mitochondrial L-lactate dehydrogenase [19,20]. In the mitochondrial matrix, pyruvate provides acetyl-CoA via the pyruvate dehydrogenase complex and oxaloacetate (OAA) involving the pyruvate carboxylase. Due to citrate synthase, pyruvate and oxaloacetate give citrate which can be exported to allow for FFA synthesis is the cytoplasm in the de novo lipogenesis (DNL). Abbreviations: ACC, acetyl-CoA carboxylase (ACC); ANT, adenine nucleotide translocator; CACT, carnitine-Acylcarnitine Transferase; CPT-1, carnitine palmitoyltransferase-1; CPT-2, carnitine palmitoyltransferase-2; DNL, de novo lipogenesis; electron transfer flavoprotein (ETF); ETFDH, ETF dehydrogenase; FFA, free fatty acids; β-HB, β-hydroxybutyrate; MPC, mitochondrial pyruvate carrier; OAA, oxaloacetate; PEP, phosphoenolpyruvate; TG, triglycerides; VLDL, very low-density lipoprotein [16,21].

This important mitochondrial pathway includes FFA β-oxidation, the tricarboxylic acid cycle (TCA), electron flow along the electron transport chain, electrochemical proton gradient generation, and ATP synthesis. In starvation, ketone bodies are produced due to absence of oxaloacetate used in gluconeogenesis. Pyruvate can enter the mitochondrion via the mitochondrial pyruvate carrier (MPC) as well as be synthesised from L-lactate after transport of L-Lactate in the matrix, via its own carrier, and oxidation via the mitochondrial L-lactate dehydrogenase [19,20]. In the matrix, pyruvate can provide Acetyl-CoA via the pyruvate dehydrogenase complex and oxaloacetate (OAA) via the pyruvate carboxylase. Due to citrate synthase, pyruvate and oxaloacetate give citrate which is exported for FFA synthesis is the cytoplasm during DNL [21]. For fatty acid catabolism, the acyl-CoA synthase transforms the cytosolic FFA into fatty acyl-CoA (Figure 1). Acyl-CoA+ carnitine are catalysed to CoA and acylcarnitine by the carnitine palmitoyl-transferase 1 (CPT-1) which is in the outer side of the inner mitochondrial membrane. Acylcarnitine can enter the mitochondria across the inner membrane in exchange with L-carnitine. This step requires the acylcarnitine/L-carnitine antiporter. The carnitine palmitoyl-transferase 2 (CPT-2), localized at the matrix side of the inner membrane, will process the acyl-carnitine to Acyl-CoA+ L-carnitine (the latter ready to be exchanged with new incoming Acyl-carnitine). The resulting acyl-CoA in the mitochondrial matrix is ultimately oxidised via the β-oxidation to acetyl-CoA which then enters the tricarboxylic acid (TCA) cycle with production of carbon dioxide and water. A further step implies the activation of the electron transport chain and ATP production. 

## 3. General Aspects of NAFLD

### 3.1. Definition

The term nonalcoholic fatty liver disease (NAFLD) points to the deposition of excess TG as lipid droplets in the cytoplasm of hepatocytes. Steatosis is defined as a hepatic TG level exceeding the 95th percentile for lean, healthy individuals (i.e., >55 mg per g of liver), histologically defined when 5% or more of the hepatocytes contain visible intracellular triglycerides [22,23] or the estimated liver fat content is ≥5% by a magnetic resonance imaging proton density fat fraction (MRI-PDFF) or ≥5.56% by magnetic resonance spectroscopy [24]. 

NAFLD has become the leading liver disease worldwide with an estimated 2 billion individuals affected [25]. NAFLD represents a spectrum of disease that may develop in individuals without significant alcohol consumption [26] and ranges from steatosis to steatohepatitis. Nonalcoholic fatty liver (NAFL), featuring simple steatosis, with little or no inflammation and no evidence of hepatocellular injury, affects about 80% of NAFLD subjects and is the non-progressive form since the risk of progression to liver cirrhosis is minimal [27]. About 20% of NAFLD manifests as nonalcoholic steatohepatitis (NASH), featuring steatosis, inflammation, and hepatocellular injury with ballooning and apoptosis. Histological findings may be indistinguishable from those of alcoholic steatohepatitis [28]. Individuals afflicted by NASH are at high risk of developing fibrosis [29,30,31,32] and NASH has an increased potential of progressing to (cryptogenic) cirrhosis and hepatocellular carcinoma (HCC) [33,34]. 

Although NAFLD is by far the most prevalent cause of liver steatosis, ectopic fat accumulation may occur in the liver for a variety of reasons in viral hepatitis B and C (in particular genotype 3), lipodystrophy, Wilson’s disease, starvation, parenteral nutrition, abetalipoproteinemia, hepatotoxic drugs (e.g., methotrexate, tamoxifen, glucocorticoids, amiodarone, valproate, and anti-retroviral agents for HIV), pregnancy, HELLP (hemolytic anemia, elevated liver enzymes, low platelet count) syndrome, Reye syndrome, and inborn errors of metabolism (i.e., lecithin-cholesterol acyltransferase deficiency, cholesterol ester storage disease, and Wolman disease). However, alcohol-associated liver injury remains the second most frequent aetiology of steatosis. The similarity and overlap between alcohol-associated liver disease and NAFLD has been the source of confusion and the subject of academic debate. The term “non-alcoholic” indeed overemphasizes “alcohol” and underemphasizes the role of metabolic risk factors, since NAFLD is commonly associated with obesity, hypertension, dyslipidaemia, and diabetes [17,26]. To acknowledge that NAFLD is no longer a diagnosis of exclusion, and it represents a continuum of liver disease caused by metabolic derangements, a change in terminology from NAFLD to metabolic dysfunction-associated fatty liver disease (MAFLD) has been recently proposed. Accordingly, hepatic steatosis is associated with at least one of the following three comorbidities: overweight/obesity (especially expansion of visceral fat), presence of type 2 diabetes mellitus, or evidence of metabolic dysregulation [35]. Nevertheless, some authors warned that understanding of the molecular basis of the disease entity, new insights in risk stratification, and other important aspects of NAFLD may be more urgent than nosology itself [36]. Indeed, there remains much to learn about the contribution of environment, comorbidities and the gut microbiome to the pathogenesis and natural history of NAFLD [16,37,38,39].

### 3.2. Prevalence and Natural History

NAFLD has become the most frequent liver disorder of our times [22,40,41,42]. The median prevalence of NAFLD is about 25% worldwide and trends are increasing [36,43,44]. This is likely due to the increasing prevalence of obesity, type 2 diabetes mellitus, sedentary lifestyles, dyslipidemia, and metabolic syndrome, mainly in North America and Europe [43,45,46,47]. However, the burden of NAFLD has also become evident in non-obese individuals (‘lean NAFLD’), with a prevalence of about 10%–30% in both Western and Eastern countries [48], typically associated with metabolic dysfunction and a comparatively increased cardiovascular risk [46,49]. NAFLD puts the population at increased risk for liver-related mortality as well as all-cause-mortality due to increased risk of cardiovascular disease and extrahepatic malignancies [50,51,52]. Liver fibrosis is currently the strongest known predictor of poor clinical outcomes in NAFLD. The time sequence of fibrosis progression in NAFL is significantly slower (average 14 years) than in NASH (about 7 years) and even less in a subgroup of ‘rapid progressors encompassing 10% to 20% of patients with NAFLD [27]. Thus, much attention has been devoted to the identification of predictors of rapid progression (i.e., higher serum ALT, morbid obesity, diabetes, and possibly genetic susceptibility with family history of cirrhosis in first-degree relatives) [53,54,55]. Once cirrhosis has developed in NAFLD, the incident risk of developing HCC is about 1.5%–2% per year. Therefore, HCC screening in NASH-related cirrhosis is recommended [56]. NAFLD is now the second leading indication for liver transplantation in the US, including a growing number of cases with NASH-related HCC [44].

### 3.3. Diagnosis

Liver biopsy followed by liver histology is the gold standard for diagnosing NAFLD. The procedure is usually echo-assisted and performed by transcutaneous puncture of the liver after local anaesthesia. A cylindric liver fragment is promptly placed in a solution containing formalin. The procedure, however, is invasive, and exposes patients to the risk of potential complications. The compliance of the patients is therefore very low. Liver biopsy should be reserved to subgroup of patients with suggestive signs/symptoms/evidence of steatohepatitis or early cirrhosis and when careful histological assessment is required to quantify the degree and stage of liver damage as fibrosis, inflammation, and necrosis, or during research protocols looking at the progression of liver fibrosis and efficacy of specific therapies.

Therefore, in clinical practice, the diagnosis of liver diseases relies on a history, physical exam and tests that investigate morphological and functional aspects. The liver is essential for many metabolic and energetic processes in the body and there is no single test that could assess liver function in a comprehensive way. Each test provides a specific set of information focusing on various mechanisms involved in liver function. A major challenge in clinical hepatology is therefore to appropriately combine the results of diagnostic tests in an accurate and complementary way to achieve the final diagnosis.

By history, NAFLD patients often carry one or more components of metabolic syndrome or “fellow travellers”, such as cholesterol cholelithiasis [57,58]. Other causes of liver steatosis and chronic liver diseases must be therefore excluded [59]. In NAFLD, alcohol consumption should be absent or very limited. This includes not more than three standard drinks/day (i.e., 21 drinks/week) in men or not more than 2 drinks/day in women (i.e., 14 drinks/week, equal to 14 g of pure alcohol/standard drink = 98 kcal), as indicated by the American Association for the Study of Liver Diseases [26]. In fact, alcohol consumption greater than the threshold puts individuals at risk of alcoholic liver disease eventually associated with coexisting NAFLD. This situation makes the diagnosis of NAFLD and risk assessment even more difficult.

Laboratory tests include serum aminotransferase levels as markers of hepatocyte cytolysis, but they are not sufficient for making the diagnosis, as laboratory tests may be normal in patients with NAFLD and may be abnormal in patients with many other conditions. Serum alanine aminotransferase (ALT) in NAFLD is typically higher than serum aspartate aminotransferase (AST) unless the disease has already progressed [60]. Serum tests to assess for other disorders include viral hepatitis serology, iron studies, and autoimmune antibody assays. Some of these ‘static’ tests measure serum parameters of synthesis (prothrombin, cholesterol, albumin), hepatocellular injury (transaminases), detoxification (ammonium), excretion and cholestasis (bilirubin, alkaline phosphatase, GGT) [61].

Imaging techniques in the evaluation of NAFLD include abdominal ultrasonography, computerized tomography (CT), and magnetic resonance.

Abdominal ultrasonography can easily detect a hyperechoic texture in the liver (“bright liver”) due to diffuse fatty infiltration. The main advantages of ultrasound include wide availability, safety, and low-cost. This non-invasive technique can easily allow a screening of patients at risk and is a useful tool for monitoring treated patients. However, liver ultrasound is not able to distinguish the necro-inflammatory changes typical of steatohepatitis, and has a poor accuracy in diagnosing the presence of a mild steatosis (i.e., <30%) [62]. Therefore, the ultimate diagnosis of both NASH and NAFLD can be underestimated.

Computed tomography can assess the liver brightness, measuring pixel values in Hounsfield Unit with quantitative determination of attenuation in comparison with the fat-free spleen [63,64]. The possibility of quantitative results is the main advantage of this imaging technique. However, as for ultrasound, the diagnostic accuracy of liver CT decreases with lesser severity of steatosis, with a sensitivity of 52–62% in case of mild steatosis (i.e., fat fraction of 10–20%) [65].

MR-based methods including proton spectroscopy and calculation of the proton-density fat fraction (PDFF) are far superior to ultrasound or CT in measuring intrahepatic fat content but, as for ultrasound and computed tomography, cannot distinguish between simple steatosis and steatohepatitis. PDFF measure, however, represents an advantage, as compared with CT, since it requires no internal calibration or reference standard. Advanced MR techniques can also consider confounders as iron overload, and can easily and rapidly allow a volumetric assessment of NAFLD [66].

By contrast, there has been significant progress in the non-invasive assessment of fibrosis in NAFLD. Vibration-controlled transient elastography is increasingly used as a point-of-care method to assess and regularly monitor fibrosis based on the liver stiffness and can also be utilized to grade hepatic steatosis. While there are additional ultrasound-based liver stiffness measurement techniques, MR elastography has proven more accurate although this method currently remains primarily in the realm of research and clinical trials due to its significant cost.

Of note, none of the imaging techniques employed to diagnose NAFLD will explore the true “dynamic” liver function and need to be integrated with further “functional” examination techniques, such as breath test.

The management of NAFLD is still a matter of debate. According to AASLD guidelines, systematic screening for NAFLD is not advisable at this time, since there is no consensus about the true cost-effectiveness of the screening [16,26]. In addition, there is no licensed or registered pharmacotherapy for NAFLD and management remains focused on healthy lifestyles as previously discussed by our group [45,46,67]. Early identification of risk factors associated with NAFLD progression is therefore paramount to delay or prevent the consequences related to advanced liver disease. However, reliable, and sensitive non-invasive diagnostic tests are still lacking in NAFLD and are actively being investigated. In this respect, diagnostic tests focusing on mitochondrial function may provide novel diagnostic and prognostic possibilities both during the evolution of disease and in therapeutical trials. These aspects are discussed in the following sections.

## 4. Mitochondrial Dysfunction in the Liver

Mitochondrial dysfunction is one of the most distinctive characteristics of NAFLD [68]. In NAFLD patients, increased plasma levels of FFA are firstly associated with increased intrahepatic inflow [24] and early mitochondrial biogenesis through peroxisome proliferator-activated receptor-α (PGC1-α) activation. This step, in turn, leads to increased FFA oxidation rates and increased or unchanged mitochondrial function [69]. Coupling of FFA oxidation to ATP generation might be dysfunctional already, because of emerging ultrastructural changes and increased expression of uncoupling proteins. With progression of NAFLD, however, mitochondrial ATP generation is further impaired resulting in defective cellular energy charge [70,71,72,73]. The precise pathways governing such changes of mitochondrial performance are still unknown.

The increased accumulation of FA in the hepatocytes (neutral lipid droplets) during insulin-resistance-associated NAFLD, which is pathologically defined as hepatic steatosis, lead to a series of mitochondrial alterations ranging between mitochondrial DNA (mtDNA) damage to sirtuin alteration. The mtDNA, a circular double-stranded molecule located in the mitochondrial matrix, encodes about the 10% of mitochondrial proteins, the others being encoded by the nuclear DNA. mtDNA encodes proteins necessary for the assembly and activity of mitochondrial respiratory complexes [74]. Ongoing oxidative stress during steatosis can severely impair mtDNA function [5] with further amplification of oxidative stress, mitochondrial biogenesis, and ultimately NAFLD severity and inflammation [75,76,77,78].

Alteration of the mitochondrial function compromises also the prooxidant/antioxidant balance, with an increase in non-metabolized fatty acids (FA) in the cytosol as a consequence of the blockade of FFA β-oxidation and the resulting stimulation of ROS production [79,80]. Mitochondrial dysfunctions are often accompanied by considerable ultrastructural changes such as megamitochondria, loss of cristae, and formation of paracrystalline inclusion bodies in the organelle matrix [81].

In addition, in NAFLD, the excessive accumulation of lipotoxic lipids in the hepatocyte generates a dysfunctional electron transfer chain with generation of abnormal levels of ROS via involvement of glycerol 3-phosphate dehydrogenase (GPDH), α-ketoglutarate dehydrogenase (AKGDH), and pyruvate dehydrogenase (PDH). Besides, the excessive accumulation of FFA into mitochondria, subsequent to an increased uptake or an insulin-resistance situation, may elicit an increase of the inner mitochondrial membrane permeability. Mitochondrial cytochrome P450 2E1 (CYP2E1), a potential direct source of ROS, has been shown to have an increased activity in a rodent model of NASH as well as in NASH patients [82,83]. CYP2E1, a cytochrome responsible for long-chain fatty acid metabolism, produces oxidative radicals and could also act as a part of the “second hit” of the pathophysiological mechanism of NAFLD [84]. In addition to the pro-oxidant mechanism, a decreased activity of several detoxifying enzymes was seen using an experimental model of NASH. Glutathione peroxidase (GPx) activity is reduced likely due to GSH depletion and impaired transport of cytosolic GSH into the mitochondrial matrix [85]. The initial mitochondrial dysfunction can be further exacerbated by the production of mtDNA mutation by ROS and highly reactive aldehydes, such as malondialdehyde (MDA) and 4-hydroxy-2-nonenal (4-HNE), through lipid peroxidation following the interaction between ROS and PUFA. Cytochrome C oxidase may be directly blocked by MDA while 4-HNE may contribute to “electron leakage” uncoupling complex 2 of the ECT whose oxidative capacity may be also diminished by derivative damage by interaction between mitochondrial membranes and both MDA and 4-HNE [86].

Aquaporin-8 (AQP8), a pleiotropic aquaporin channel [87,88,89] allowing movement of hydrogen peroxide in addition to water and ammonia, localized at multiple subcellular levels in hepatocytes [90], is also present in mitochondria, where it has been suggested to facilitate the release of hydrogen peroxide across the inner mitochondrial membrane following ROS production [91].

Mitochondrial redox imbalance and high Ca^2+^ uptake have been shown to induce the opening of the permeability transition pore (PTP) with consequent disruption of energy-linked mitochondrial functions and triggering of cell death in many disease states including non-alcoholic fatty liver disorders [92].

**Figure 2:**
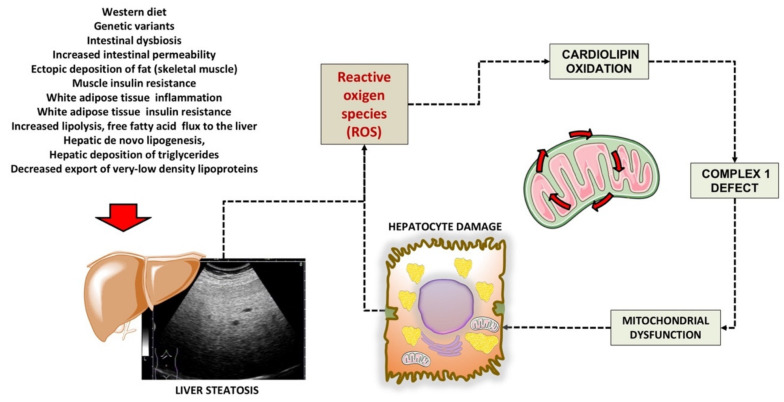
Putative mechanisms of damage involving cardiolipin in liver mitochondria during liver steatosis. Following several predisposing factors, liver steatosis develops. The increased production of ROS is associated with mitochondrial cardiolipin oxidation, defective complex 1, and furthers mitochondrial and hepatocyte dysfunction. Cardiolipin is a phospholipid localized almost exclusively within the inner mitochondrial membrane close to complexes I and III of the mitochondrial respiratory chain. Mechanisms of damage have been elucidated in the study by Petrosillo et al., using the rodent model fed a choline-deficient diet to induce simple liver steatosis [94].

In previous studies, we used the rat model of a choline-deprived diet for 30 days inducing simple liver steatosis. In particular, peroxidation of the membrane lipid components participates in mechanisms of oxygen-free radical toxicity [93]. Cardiolipin is a phospholipid localized almost exclusively within the inner mitochondrial membrane close complexes I and III of the mitochondrial respiratory chain. Notably, cardiolipin becomes an early target of oxygen-free radical attack, a step leading to deranged mitochondrial bioenergetics. In a first study, we assessed various parameters related to mitochondrial function such as complex I activity, oxygen consumption, reactive oxygen species (ROS) generation and cardiolipin content and oxidation. Complex I decreased by 35% in mitochondria isolated from steatotic livers, compared with the controls, and changes were associated with parallel changes in state 3 respiration. At the same time, hydrogen peroxide (H2O2) generation increased significantly in mitochondria. The mitochondrial content of cardiolipin, a phospholipid required for optimal activity of complex I, decreased by 38% in parallel with an increase in the level of peroxidised cardiolipin. Data confirm that dietary steatosis induces mitochondrial dysfunction revealed by deranged complex I function attributed to ROS-induced cardiolipin oxidation and function [94]. A putative scenario of damage is depicted in Figure 2.

In addition, using the choline-deficient steatogenic diet in the rat model, we measured the circulating and hepatic redox active and nitrogen-regulating molecules thioredoxin, glutathione, protein thiols (PSH), mixed disulphides (PSSG), NO metabolites nitrosothiols, nitrite plus nitrate (NOx), and lipid peroxides (TBARs). The histologically proven hepatocellular steatosis (75% of liver weight at day 30) was paralleled by increased serum and hepatic TBARs (r = 0.87, *p* < 0.001) and lipid content (r = 0.90, *p* < 0.001). Liver glutathione and thioredoxin 1 initially increased and then decreased, while, from Day 14, PSH decreased, and NO derivatives increased. Mitochondrial nitrosothiols were inversely related to thioredoxin 2. These results suggest that adipocytic transformation of hepatocytes is accompanied by major interrelated modifications of redox parameters and NO metabolism, especially at the mitochondrial level, suggesting an early adaptive protective response but also an increased predisposition towards pro-oxidant insults [95].

The combination of these events explains how mitochondrial dysfunction becomes a key step paving the way to cells and organ damage. The main events in this scenario include the lack of energy supply by ATP and excessive generation of ROS. In case of prolonged starvation or diabetes, for example, ketone body synthesis occurs, when oxaloacetate is depleted due to its involvement in gluconeogenesis. In this scenario in the mitochondria, the acetyl-CoA does not enter the TCA cycle, and is converted to ketone bodies (i.e., acetone, acetoacetate, and β-hydroxybutyrate (β-HB)). Some metabolic markers might appear in the systemic circulation, for example with an abnormal acetoacetate/β-OH-butyrate ratio [9,96], but they cannot be easily monitored and are rather unspecific. In type 2 diabetes mellitus and NAFLD, the hepatic mitochondrial metabolism is impaired [97,98], associated with remodelling of mitochondrial lipids [99], and increased mitochondrial mass and respiratory capacity [100]. Lipotoxicity can influence acetyl-CoA metabolism [101] with excessive turnover of the tricarboxylic acid cycle [97]. In the steatogenic model of cultured hepatocytes, the combination of fructose and FFA caused profound effects on the lipogenic pathways. We noticed increased steatosis and reduced cell viability, increased apoptosis, oxidative stress and, mitochondrial respiration in the Seahorse system. Hepatic cell abnormalities can be prevented, and in this model, the damage improved by treating the cells with the nutraceutical silybin [102].

Mitochondrial dysfunction is associated, in NASH, with the ongoing oxidative state of hepatocytes, and is able to affect intracellular signalling pathways by generation of DAMPs and to activate stellate cell [103].

A recent in vitro study demonstrated that circulating factors contained in plasma samples from NAFLD patients were able to generate a NAFLD-like phenotype in isolated hepatocytes, with effects mediated by NLRP3-inflammasome pathways and by the activation of intracellular signalling related to SREBP-1c, PPAR-γ, NF-kB and NOX2 [104].

Besides external conditions affecting the metabolic homeostasis and mitochondrial function, genetically driven conditions can also lead to altered hepatic mitochondrial activity and peroxisomal β-oxidation [105,106,107]. In particular, the membrane bound O-acyltransferase domain containing 7-trans-membrane channel-like 4 (MBOAT7-TMC4) is localized to the intracellular membranes of mitochondria, endoplasmic reticulum, and lipid droplets. MBOAT7-TMC4 acts as lysophospholipid acyltransferase, and regulates the incorporation of arachidonic acid into phosphatidylinositol [108]. This pathway, due to its key role, might be considered a promising therapeutic target. The expression of MBOAT7-TMC4 is decreased in the rs641738 polymorphism, and this leads to the onset of liver steatosis and to an altered liver histology [109,110], to fibrosis in alcoholic liver disease [111] and in chronic hepatitis C [112]. In a mice model of NASH, the deletion of hepatocyte Mboat7 is linked to with increased fibrosis, with no effects on inflammation [113]. Mboat7 also promotes the degradation of lysophosphatidylinositol, and the accumulation of this molecule in Mboat7 KO mice generates NASH [114], also through the activation of the G-protein coupled receptor GPR55 [115]. Aging processes is associated with altered subcutaneous adipose tissue function, with mechanisms that involve a reduced mitochondrial activity [116,117], the accumulation of senescent adipocytes, and impaired development of pre-adipocytes [118]. Liver mitochondria also play a relevant role in lipid-induced hepatic insulin resistance, through mechanisms linking specific lipid metabolites and cellular compartments and leading to subcellular dysfunctions [119]. In particular, the quantitative assessment of DAG stereoisomers (sn-1,2-DAGs, sn-2,3- DAGs, and sn-1,3-DAGs) and ceramides in the endoplasmic reticulum, mitochondria, plasma membrane, lipid droplets, and cytosol showed, using an antisense oligonucleotide, the onset of hepatic insulin resistance in rats, which was associated with the acute liver-specific knockdown of diacylglycerol acyltransferase-2. The dysregulation of peroxisome proliferator-activated receptor-gamma co-activator-1α (PGC-1α) contributes to the pathogenesis and to the sequence of NASH-HCC, with metabolic pathways involving gluconeogenesis, fatty acid oxidation, antioxidant response, DNL, and mitochondrial biogenesis [120].

Experimental data indicate that mitochondrial dysfunction is also a specific target for toxic chemicals of environmental origin mainly introduced bycontaminated food and water and leading to NAFLD.

A recent study in 2446 young adults showed that toenail cadmium concentration, a marker of long-term exposure, was associated with higher odds of prevalent NAFLD independently from race, sex, BMI or smoking status [121]. In a mouse model of chronic cadmium exposure, hepatic Cd concentrations ranging from 0.95 to 6.04 μg/g wet weight were able to induce, following a 20-week exposure, NAFLD and NASH like phenotypes linked with mitochondrial dysfunction, fatty acid oxidation deficiency and a significant suppression of sirtuin 1 signalling pathway [122]. Epidemiologic studies point to a positive association between arsenic exposure (i.e., urinary arsenic concentrations) and risk of NAFLD [123]. This evidence is paralleled by experimental findings showing, in isolated rat liver mitochondria exposed to arsenic, a marked decrease in total mitochondrial dehydrogenase activity with increased ROS generation, MMP, and MDA levels, and decreased activity of mitochondrial catalase and GSH [124].

In a cohort of 6389 adolescents from the NHANES survey, blood mercury levels were linked with the risk of NAFLD, with the most evident association in underweight or normal weight subjects [125]. In a recent animal model, exposure to methylmercury during 12 weeks induced mitochondrial swelling, ROS overproduction, increased gluthatione oxidation, and reduced protein thiol content [126].

Similar pathways linking environmental pollution with NAFLD in terms of both epidemiologic findings of increased NAFLD risk and animal/in vitro evidence of mitochondrial dysfunction also have been shown in the case of air pollution [127,128,129], endocrine disrupting chemicals [127,128,129,130,131], and pesticides [132,133,134,135].

There are few ways to investigate mitochondrial metabolic processes, i.e., using isolated organelles, mitochondrial fractions, and cell culture [136]. Few studies explored the impaired mitochondrial function in NAFLD. Protocols investigating the effects of xenobiotics and drugs on mitochondrial function can provide some information [137,138]. Metabolomics can also explore specific mitochondrial functions [139] by studying genetic perturbations [140]. The measure of circulating mitochondrial DNA (mtDNA) is another biomarker of mitochondrial dysfunction. Changes to liver mitochondrial DNA (mtDNA) can precede mitochondrial dysfunction and irreversible liver damage. Malik et al. [141] by using a rodent dietary approach, demonstrated that a high-fat or a high-fat/high-sugar diet for 16 weeks was associated with fast alterations in mtDNA. Thus, dietary changes in liver mtDNA can occur in a relatively short time. Mouse liver contained a high mtDNA content (3617 +/− 233 copies per cell), which significantly increased when the mice were fed an HFD diet. This increase, however, was not functional; i.e., it was not translated into an increased expression of mitochondrial proteins. Furthermore, liver dysfunction was accelerated alongside the downregulation of mitochondrial oxidative phosphorylation (OXPHOS) and mtDNA replication machinery as well as upregulation of the mtDNA-induced inflammatory pathways.

## 5. Studying Liver Mitochondrial Function at a Translational Level

Strategies to diagnose mitochondrial damage and afterwards to prevent progression by therapy in NAFLD are actively being investigated. All the above-mentioned procedures focusing on mitochondrial function, however, lack true translational value, can be complex, expensive, and make the comparison between different models and in vivo studies somewhat difficult. A major problem is that mitochondria function at the crossroads of several complex metabolic processes, which can be influenced by unknown precursors affecting metabolic pathways.

Thus, no specific, easily available test provides information on liver mitochondrial status in clinical medicine, and we miss reliable biomarkers that inform on hepatocyte mitochondrial function and fitness in the NAFLD. Further studies and novel diagnostic tools are required in this field. The following paragraphs will focus on the use of breath tests by stable isotopes to explore liver mitochondrial function.

## 6. General Features of BT

BT represent an expanding field in diagnosis of liver function and are “dynamic” tools dealing with distinct functional aspects of liver metabolism. BTs can be employed for the follow-up of liver disease, including mitochondrial function in NAFLD [96]. Liver BT can provide information about the complex metabolic function of the liver by marking a given substrate with the stable isotope ^13^C, measured in breath as ^13^CO_2_. Our group has provided studies dealing with evaluation of both mitochondrial and microsomal activity in the liver [36,96,142,143,144,145,146,147,148,149]. The rationale of BT depends on a given substrate that is metabolized at different levels in the body. The metabolized substrate produces gases (e.g., CO_2_, H2) transferred to blood, excreted, and quantified in expired air. Various sensors can detect the end-product in breath. The measured metabolite becomes the biomarker of a specific metabolic process [150]. Examples of BTs include the urea BT for the diagnosis of infection by *H. pylori* in the stomach, the hydrogen breath test for the diagnosis of lactose intolerance, or the study of small intestinal bacterial overgrowth [151,152,153,154]. A few BTs are relatively simple to perform, safe, and non-invasive, with potential applications in several conditions. Liver BTs are used to assess the hepatocyte capacity to metabolize a substrate in a time-dependent way [144,155]. Few BT have been developed and employ substrates labelled with the stable isotope (non-radioactive), naturally occurring ^13^C marking one specific carbon atom in the substratum. The essential characteristics of ^13^C-BT when assessing liver function are depicted in Table 1 [143,156].

A single functional test cannot explore the whole liver function, since liver metabolic pathways are characterized by intrinsic complexity in terms of uptake, site of metabolization, and pathways involved during the hepatic phase of the substrate.

**Figure 3:**
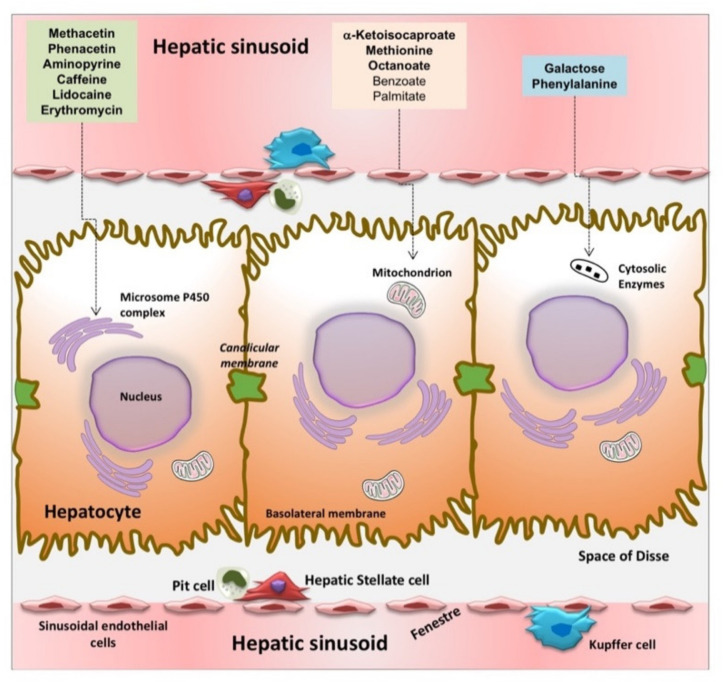
Structure of the hepatic sinusoid and types of substrates used for breath test analysis of liver function. The hepatic sinusoid represents a unique, dynamic microvascular structure. It serves as the principal site of exchange between the blood, and the space of Disse (perisinusoidal space). The main nonparenchymal cells populating the sinusoid are the fenestrated sinusoidal endothelial cells in contact with the blood, the phagocytic Kupffer cells, which adhere on the luminal aspect, and the hepatic stellate cells which are specialized pericytes that extend processes throughout the space of Disse. They serve as myofibroblasts during times of hepatic injury and repair, liver-associated lymphocytes (Pit cells). This mass of sinusoidal nonparenchymal cells account for approximately 6% of the total liver volume, and about 30% of the total number of liver cells. At the liver sinusoid, several substrates are used to explore liver function by a breath test (see text for details) [143].

After proximal intestinal absorption, the substrate reaches the liver via the portal vein, and undergoes metabolism in the hepatocyte with ultimate production of ^13^CO_2_, which appears quickly in expired air [96,143,157]. For liver function, several substrates have been developed and marked at one carbon site with the natural stable isotope carbon ^13^C. Substrates are designed to target liver microsomes (i.e., methacetin, aminopyrine, phenacetin, caffeine, lidocaine, and erythromycin), cytosolic enzymatic activity (i.e., phenylalanine and galactose), and mitochondria (methionine, KICA, and octanoic acid) (Figure 3).

Stable isotopes BT can be used to assess the course of a disease or the effect of therapies and has no restrictions with respect to infants and pregnant women. Few drawbacks are the limited availability and costs of the equipment, costs of substrates, and need for experienced operators. To date, the application of stable isotope breath testing has remained rather experimental in few referral centres and currently no substrate (apart from urea for the diagnosis of *H. pylori* infection) is officially approved for clinical use [96,147,158]. Approval from local ethical boards is recommended [96].

### 6.1. Methodology of ^13^C-BT

**Figure 4:**
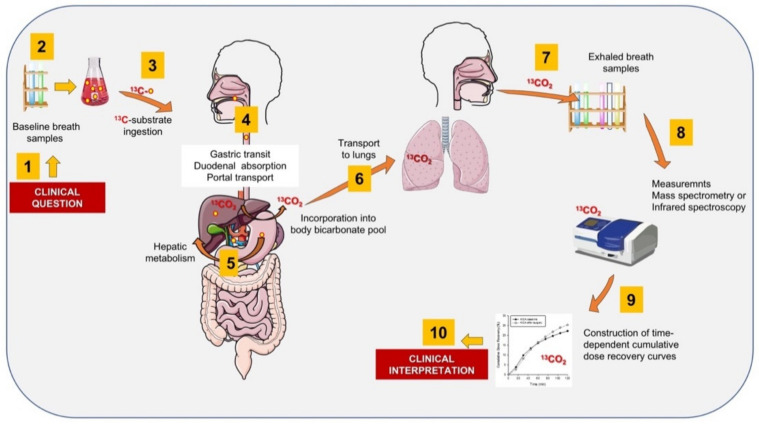
The ten-step protocol for the breath analysis using ^13^C-substrates for the study of liver function. Starting from the clinical question (1), the fasting subject collects breath samples (2), and, after ingestion of the solution containing the substrate (3), further steps include gastric emptying, duodenal absorption, and portal transfer to the liver (4). The substrate undergoes metabolization at different levels (i.e., microsomes, cytosol, and mitochondria) with production of ^13^CO_2_ (5). Afterwards, ^13^CO_2_ undergoes incorporation into the bicarbonate pool and quick transport to the lung (6) where appears in exhaled air ready for collection (7). Breath samples are measured by infrared spectroscopy or mass spectrometry (8) with software constructing time-dependent curves of the metabolic process (9). The information serves to elaborate the specific clinical interpretation of liver function (10). An important assumption is that gastric emptying is not severely delayed and that the lung function is not severely impaired [96,143,145,158]. Cartoon adapted from Di Ciaula et al., *Eur. J. Internal Medicine* 2021 [39].

Figure 4 depicts the general methodology of ^13^C-BT. Briefly, the substrate labelled with ^13^C is dissolved in tap water and administered by oral route [96,158]. Subjects are required to be fast overnight, i.e., at least 8–12 h. No special diet is required the day before the test, which is generally performed in the morning.

The test is performed in a quiet room and the subject should not exercise and should refrain from smoking for at least 30 min before and during the test. This approach will minimize variations in endogenous CO_2_ production due to physical activity or combustion. A first breath collection is performed at baseline into plastic bags (250–500 mL) or special glass tubes with rubber caps (exetainer) that are properly labelled. Afterwards, the subject drinks the solution with substrate within 1–3 min. Samples of expired air are then collected at different time points, usually every 15 min up to 30–120 min, depending on the protocol. As an example, a total of 9 samples are taken for a 15-min sampling for 2 h (i.e., at time 0, 15, 30, 45, 60, 75, 90, 105, and 120 min). Plastic bags are preferred if few samples/subjects are necessary to study, while exetainers are preferred if several samples are planned on the same day or the automatic sampling system is available. Breath test can be also defined as “field tests” since with the subject appropriately instructed, samples can be collected at home, in the ward or in the outpatient clinics simultaneously and centralized in the referral lab. Bags and exetainers are tightly closed and samples are measured within 24–48 h, where the equipment is available.

The enrichment of expired ^13^CO_2_ is then analysed by isotope ratio mass spectrometry or by infrared spectroscopy (e.g, IR-300 plus, Beijing Richen-Force Science & Technology Co., Ltd., Bejing, China or Helifan Plus, Fischer ANalysen Instrumente GmbH, Leipzig, Germany). Devices based on molecular correlation spectroscopy can detect variations less than 1:1000 in the ^13^CO_2_/^12^CO_2_ ratio.

The equipment is set to calculate the rate of exhalation of ^13^CO_2_ at each time point from the measured increment in the isotopic abundance of ^13^CO_2_ (δ^13^CPDB). The algorithm takes into account the purity of the labelled compound with a constant endogenous production of CO_2_ of 300 mmol/m2/h. The results are expressed as a percentage of the administered dose recovered per hour. The cumulative percentage of ^13^CO_2_ in breath is calculated as the area under curve (AUC) of the ^13^CO_2_ exhalation rate compared with the time curve determined by linear interpolation using the trapezoidal rule [159,160]. For the ^13^C-methacetin breath test, the authors reported that the inter-test coefficient of variation of the ^13^C-MBT in healthy volunteers and liver disease patients is 13.2% for the PDR peak, with a coefficient of variation of 23.9% for the cumulative PDR at 20 min cPDR20.

### 6.2. Factors Potentially Affecting the Use of ^13^C Breath Tests for the Assessment of Liver Function

The assessment of liver function by ^13^C-breath test can be affected by physiologic or pathologic conditions acting on the individual baseline CO_2_ production or on the perfusion of the liver by blood [161]. Furthermore, the liver metabolism of oral-ingested substrates can be either decreased or increased by concomitant treatments with drugs influencing the activity of the cytochrome P450 (Table 2).

A severely delayed gastric emptying (e.g., diabetic, or idiopathic gastroparesis, severe motility defects, inflammation, and malignancies) might interfere with the delivery of the substrate to the duodenum. Thus, before the examination, the operator should investigate the clinical history to detect symptoms potentially related with altered gastric emptying or conditions affecting the absorption of the substrate from the gastrointestinal tract [161,162]. In this case, the intravenous administration of the substrate (as the LiMAx^®^ test) can be useful to obtain accurate results, also limiting inter-individual variations in substrate absorption after oral administration [163,164,165,166].

### 6.3. Assessing Liver Mitochondrial Function by BT

**Figure 5:**
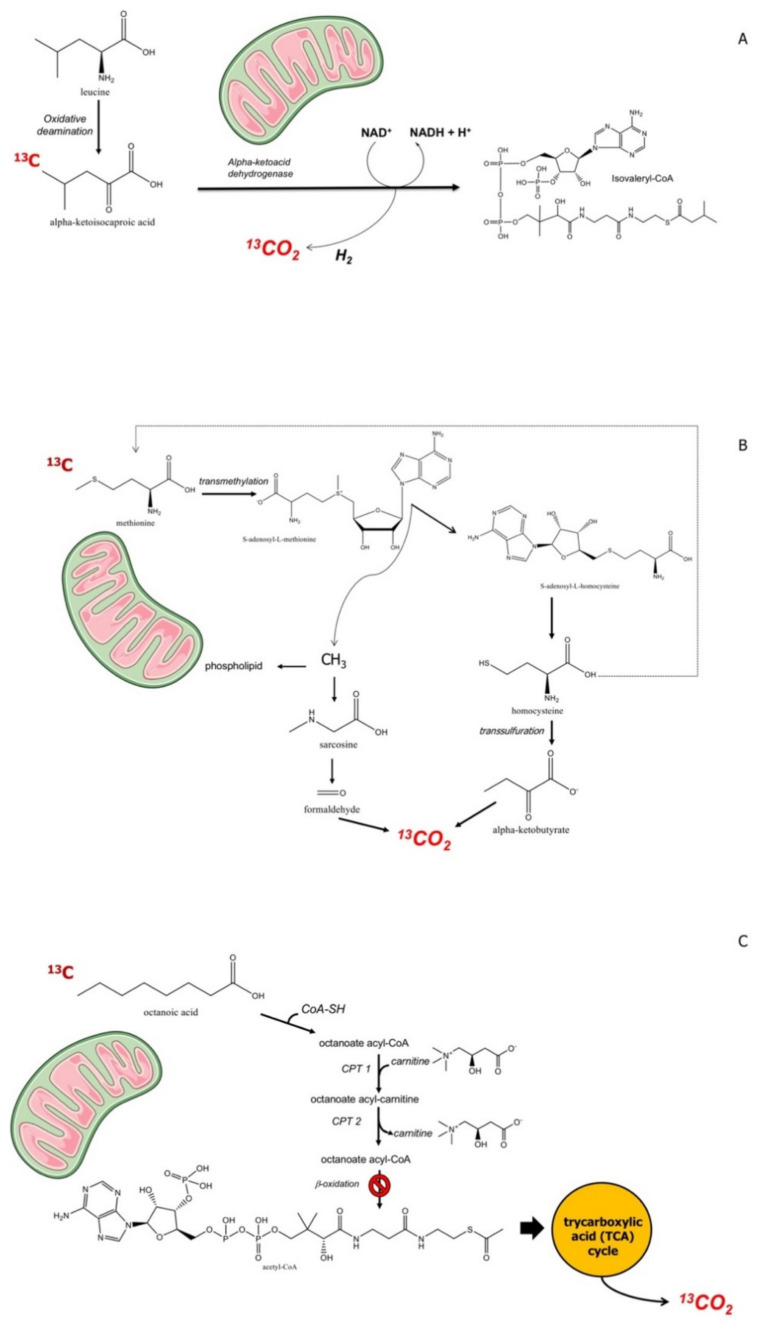
The figure shows the mitochondrial metabolism of alpha-ketoisocaproic acid (**A**), methionine (**B**), and octanoic acid (**C**). The ^13^C-enriched substrates become donors of ^13^CO_2_ which is promptly transported to the lung and excreted in breath.

The assessment of liver function and responses to therapy is often required at an early stage in clinical medicine. The assessment of early involvement of mitochondrial function could be part of this procedure adding prognostic information [167]. There are substrates that target mitochondria and can be marked with ^13^C, which becomes detectable in expired air. Substrates are alpha-ketoisocaproic acid (KICA), methionine, and octanoic acid [147]. Benzoic acid undergoes glycine conjugation, but was used only in the animal model of liver cirrhosis [168]. Following the mitochondrial catabolism of a certain substrate, CO_2_ is produced. The labelled carbon (*C) will appear in breath as *CO_2_, as a marker of mitochondrial clearance of the substrate [96]. The radioactive isotope 14C had limitations because of radiation exposure, and thus could not be used in pregnant women and children. ^13^C, by contrast, is a stable, nonradioactive, naturally occurring isotope. It accounts for about 1.1% of all the natural carbon on Earth and in plants and the food chain. The following assumption define this type of breath test, or a given exogenous substrate, i.e., Hepatic clearance = Hepatic Perfusion x Hepatic Extraction (where Hepatic Extraction is the ratio of the difference between inflow and outflow concentration ÷ by inflow concentration of the probe) [169]. The hepatic clearance is either flow-limited (range 0.7–1.0) or enzyme-limited (<0.3) [170]. Few ^13^C-labeled substrates are suitable to explore mitochondrial liver function, and include alpha-ketoisocaproic acid, methionine, and octanoic acid (Figure 3). The mitochondrial metabolism of these three substrates is depicted in Figure 5.

KICA is a substrate for branched chain alpha ketoacid dehydrogenase, located in the hepatic mitochondrial matrix [171]. Methionine is a substrate for protein synthesis or alternatively enters the methionine cycle, with subsequent transformation in S-adenosylmethionine, a methyl donor. S-adenosylhomocysteine, resulting from donation of the methyl group, is hydrolyzed to homocysteine, which, in turn, is destinated to trans-sulfuration or to remethylation to methionine. The trans-sulfuration step, in particular, generates α-ketobutyrate, which enters mitochondria with subsequent decarboxylatation [172]. Octanoic acid, on the other hand, is β-oxidized with generation of acetyl coenzyme A, which enters the Krebs cycle and undergoes oxidation to CO_2_ [172].

All these three substrates have been used to accurately assess hepatic mitochondrial function in NAFLD patients. By different substrates, BT can also allow to explore the hepatocyte at a cytosolic (^13^C-phenylalanine and galactose) or microsomal level (aminopyrine, phenacetin, caffeine, lidocaine, methacetin, and erythromycin) [172]. From a clinical point of view, these substrates provide information in different clinical settings (Table 3).

### 6.4. Potential Clinical Application

Each substrate has possible clinical applications (Table 3), as shown in patients with alcohol-related liver disease, NAFLD, viral hepatitis, liver cirrhosis, hepatocellular carcinoma, and evaluation of drug or alcohol toxicity.

#### 6.4.1. ^13^C-KICA BT

KICA has a molecular weight of 130.141800 g/mol (MF: C6H10O3, IUPAC name: 4-methyl-2-oxopentanoic acid), and is part of the metabolic pathway of the amino acid leucine (Figure 5A). The biotransformation of KICA to isovaleryl-CoA depends on the alpha-ketoacid dehydrogenase in the liver. The decarboxylation of KICA is a specific function of mitochondria, as confirmed in isolated mitochondria [182], in experimental models [157], and in humans. No gender difference exists when interpreting the results according to body composition [183]. The major competing pathway for KICA elimination is the transamination of KICA to leucine. The ^13^C-BT requires the concomitant administration of unlabelled leucine which suppresses this pathway, and this step makes the KICA BT highly sensitive for mitochondrial function. In addition, KICA decarboxylation is depends on the availability of NADH. Thus, ethanol at 0.5 g/kg body weight increases the availability of NADH (leading to decreased KICA decarboxylation), while 1 g of aspirin decreases liver NADH (leading to a higher KICA decarboxylation rate) [171]. These aspects need to be considered during the test performance and the evaluation of results. ^13^C-KICA is given at a dose of 1 mg/kg body weight plus 1 g unlabeled L-leucine while ^13^C-methacetin is given at a dose of 1.5 mg/kg body weight (generally 75 mg). The substrate is generally flavorless and dissolved in 100mL of tap water. Such a small volume shortens the drinking time and will allow the prompt initiation of the gastric emptying process.

**Figure 6:**
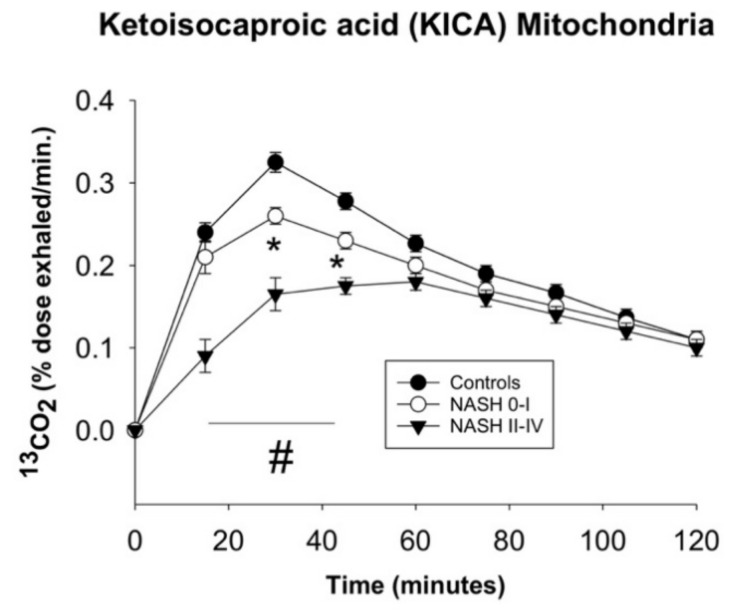
Metabolism of ^13^C-ketoisocaproic acid (KICA) as a breath biomarker of mitochondrial function. This study was conducted on 39 (20 lean and 19 obese) hypertransaminasemic patients with histologically-proven NAFLD ranging from simple steatosis (NASH 0-I) to severe steatohepatitis and fibrosis (NASH II-IV). Control subjects were 20 lean and 8 overweight healthy individuals. Compared with healthy subjects and patients with simple steatosis, NASH patients had enhanced methacetin demethylation (*p* = 0.001), but ketoisocaproate decarboxylation was mildly or greatly decreased and delayed in simple steatosis and steatohepatitis, respectively. Ketoisocaproate decarboxylation was impaired further in obese patients with NASH, but not in patients with simple steatosis and in overweight controls. Symbols (*, #) indicate significant differences compared to the controls (0.006 < *p* <0.001). From Portincasa et al. [147].

Several studies are available with KICA. The decarboxylation decreases in alcoholics compared with patients with NAFLD and controls [173,184]. In another study 13 male patients with heavy intake of alcohol during the last month were compared with 10 healthy volunteers. Abnormal liver status was confirmed by abnormal aspartate aminotransferase, alanine aminotransferase, or gamma-glutamyltransferase analyses. Healthy women had a higher percentage exhalation of ^13^CO_2_ than both healthy males and alcoholic males. Surprisingly, there was no significant impairment of KICA decarboxylation as an effect of chronic intake of alcohol or alcohol-induced steatosis. The information obtained by ^13^C-KICA are limited in case of excessive alcohol consumption likely due to a fast normalization of the values [185]. We found that KICA decarboxylation was defective in patients with histologically-proven advanced nonalcoholic steatohepatitis (NASH) but not in patients with simple steatosis [147] (Figure 6). Data were inversely related to the extent of fibrosis especially in obese patients.

In a subsequent study, we found that KICA decarboxylation was decreased in cirrhotic patients with HCC compared with cirrhotic patients without HCC and identical Child—Pugh scores [158], i.e., a classification ranging from score 5 to 15 which incorporates five variables (serum albumin and bilirubin, ascites, encephalopathy, and coagulation as prothrombin time) for assessing the prognosis of liver cirrhosis [186]. The mitochondrial function was further impaired during with radiofrequency ablation (RFA) and trans-arterial chemoembolization (TACE). The application of the ^13^C-KICA BT might extend to other conditions, since we described a slight mitochondrial malfunction in a young patient diagnosed with massive liver echinococcosis occupying most of the liver. We detected a cumulative dose recovery (CPDR) of 22% (normal: CPDR within 120 min ≥ 23% normal). The ^13^C-methacetin breath test investigating the liver microsomal function was normal. Notably, mitochondrial liver function improved the following pericystectomy and limited hepatectomy. Other applications of KICA BT are possible. Several drugs can enter the mitochondria and accumulate, a step often interfering with respiratory complexes or electron transfer [187]. For example, aspirin, ibuprofen (nonsteroidal anti-inflammatory drugs), amiodarone (antiarrhythmic agent), and valproate (an anticonvulsant, histone deacetylase inhibitor) inhibit mitochondrial fatty acid β-oxidation [187,188]. The nucleoside analogues are widely used in transplanted patients or in HIV- and HBV-infected subjects. Drugs can incorporate into mitochondrial DNA, inhibit c-DNA polymerase, and hinder the replication process [189]. Nevertheless, viral infection itself (HIV and HCV) may impair mitochondrial function, as confirmed in HCV-infected cells [190] and in patients [177]. In addition, xenobiotics can cause excessive activation of the mitochondrial permeability of the transition pores and alter mitochondrial function. Drugs include acetaminophen [191], N-nitrosofenfluramine [192], salicylate [193], and nimesulide (in vitro) [194]. The KICA BT might provide helpful results to study the integrity of liver organelles before the administration of potentially toxic drugs and to detect drug-induced mitochondrial damage before the appearance of symptoms to timely manage patients and prevent adverse effects. Examples are tacrolimus, aspirin [171], and ergot alkaloids [195].

#### 6.4.2. ^13^C-Methionine BT

Methionine has a molecular weight of 149.21134 g/mol, MF: C5H11NO2S, IUPAC name: (2S)-2-amino-4-methylsulfanylbutanoic acid. It is an essential amino acid that is involved in metabolic processes. Exogenous methionine contributes to protein synthesis [196]. Methionine can be transformed into S-adenosylmethionine, the main biological methyl donor, which is hydrolysed to homocysteine, either undergoing trans-sulfuration to α-Ketobutyrate or remethylation to methionine. α-Ketobutyrate enters the mitochondria and undergoes decarboxylation. The administration of oral L-(1-^13^C)-methionine will then be associated with production of labelled ^13^CO_2_. Transmethylation to S-adenosylmethionine also provides the substrate for the synthesis of sarcosine, which is oxidized to formaldehyde and production of CO_2_ in mitochondria. Methionine differentially labelled in the methyl group and in position 1 can be used to study the complex metabolism of methionine [175]. For mitochondrial function studies, suitable substrates are either L-(1-^13^C) methionine or (methyl-^13^C)-methionine. There are some limitations, e.g., comparisons of different studies can be quite difficult since more than one ^13^CO_2_ is formed according to the L-(1-^13^C) or (methyl-^13^C)-labelled methionine [197]. Some studies used intravenous rather than oral methionine and in this case the comparisons become even more difficult [198]. Methionine BT can provide information about mitochondrial function during acute intoxication and in chronic liver diseases. However, acute ethanol consumption impairs ^13^C-methionine decarboxylation in normal liver [174]. The metabolism of methionine decreases in patients with liver cirrhosis and especially in those with an aethanol etiology [175], in patients with biopsy-proven severe NAFLD in relation to the extent of steatosis [178], and in patients taking high-dose valproic acid [178] or nucleoside analogues for the treatment of HIV [189]. Methionine BT is reported in hepatitis C-infected cells [190], and in patients with Friedreich ataxia [179] an autosomal recessive degenerative disorder caused by loss of function mutations in the frataxin gene (FXN gene), located on chromosome 9q13 [199].

#### 6.4.3. ^13^C-octanoate BT

Octanoic acid (OA) has a molecular weight of 144.21144 g/mol, MF: C8H16O2, IUPAC name: octanoic acid, caprylic acid. OA is a straight medium chain saturated fatty acid with an 8-carbon backbone, it is found in the milk of various mammals and is a minor component of coconut oil and palm kernel oil. OA enters mitochondria independently of the carnitine transport system and is β-oxidized to acetyl coenzyme A (AcCoA) [200]. AcCoA enters the Krebs cycle and is oxidized to CO_2_ unless utilized for the synthesis of other energy-rich compounds (Figure 5C). The ^13^C-octanoate should also reflect hepatic mitochondrial function (β-oxidation capacity). For use in humans the test requires informed consent. In the animal models 13-octanoate BT was informative about liver function in rat models of acute hepatitis and thioacetamide-induced liver cirrhosis, but not in cholestatic liver injury [201]. In NASH patients, the oxidation of octanoate was either unchanged, although greater in women than men [181] or increased [180], and unchanged in those with early stage and advanced cirrhosis with and without a porto-systemic shunt [202]. Such apparently discrepant results with octanoate might be due to subtle differences in the metabolic pathways, the substrates employed, or by extra-hepatic mitochondrial oxidation of octanoate. Gender differences should be also taken into account, when considering the study of Schneider et al. [181], for example. Unfortunately, a comparison of different substrates and BT in the same group of subjects/patients has not been performed, so far. If liver damage is absent, the ^13^C-octanoate BT is a useful diagnostic test to measure the rate of gastric emptying to solids, i.e., a muffin enriched with the labelled substrate [146,203].

## 7. Why Studying Liver Mitochondrial Function in NAFLD

There is no established therapy not as a monotherapy nor in combination with NAFLD. The complexity and the number of pathogenic mechanisms involved in the full spectrum of NAFLD, makes this goal difficult to achieve and experiment with, so far [16]. Nevertheless, there might be some arguments for studying mitochondrial function in NAFLD patients (Table 4).

In general, a modification in lifestyle (i.e., diet and regular physical exercise [45,46]) and other general measures serve to maintain body weight or reduce body weight in overweigh/obese subjects. Ideally, weight loss should be in the range of 5–7% and 7–10% in NAFLD and NASH, respectively, in both overweight and obese patients [45]. This approach can improve liver biochemical tests, liver histology, serum insulin levels, and quality of life [204,205,206,207,208,209]. We have learnt that in NASH, liver fibrosis can improve after at least 10% weight reduction, although this goal is difficult to achieve in the majority of patients and to maintain for a long time [142,207]. To improve insulin sensitivity and reduce body weight, the diet must be based on long-term caloric restriction rather than intermittent fasting [210]. This approach will prevent oxidative damage [211,212]. Bariatric surgery is indicated in the subgroup of morbid obese patients or obese patients with increased cardiovascular risk. This choice can reduce the prevalence of NASH [213,214]. Specific risk factors for cardiovascular disease, diabetes mellitus must be screened and appropriately treated in NAFLD patients (e.g., antidiabetic agents, lipid-lowering therapy). Alcohol consumption, even in small amounts, is not recommended, since it is associated with progression of liver fibrosis [215].

When considering the aspects related to liver mitochondria in NAFLD, potential targets include nuclear receptors and compounds involved in different signaling pathways, mitochondrial transporters, enzymes playing a major role in mitochondrial metabolism, biomolecules involved in pathways controlling reactive oxygen species (ROS) and oxidative stress. Therapeutic strategies, however, are highly experimental and in several cases tried in animal or in vitro models. Although potentially able to ameliorate mitochondrial function in NAFLD, these agents require further evidence about use in humans, safety, efficacy, duration of treatment, type of steatosis, etc.

A moderately hypocaloric diet plus physical exercise might improve mitochondrial structure and function and alleviate inflammation [216,217,218,219]. Mitochondrial permeability transition [220,221] and mitochondrial integrity and function can improve and become more resistant to stress [221]. The exercise will decrease the insulin resistance status while increasing the hepatic mitochondrial oxidative capacity associated with increased FFA oxidation and decreased FA-derived ceramide and diacylglycerol synthesis [23,222]. With all limitations previously discussed, other options include as following: Bile acids, such as obeticholic acid [223,224,225] and ursodeoxycholic acid [226]. Agents acting as antioxidants, on nuclear receptors or mitochondrial metabolism, such as Vitamin E (α-Tocopherol) [26], Tempol [227], Resveratrol [228,229,230,231], Mitoquinone (Mito-Q) and Mitovitamin E (MitoVit-E) [232,233,234], Silymarin (major component is Silybin) [136,235,236], Corilagin [237], Anthocyanins (i.e., Cyanidin) [238,239], Dihydromyricetin [240], Berberine [241], Hydroxytyrosol [242], Cysteamine [243,244], Pentoxifilline [245,246,247], Avocado oil [248,249,250], and Pegbelfermin (via FGF21R beta) [251]. Antidiabetic drugs including Elafibranor [252,253], Liraglutide [254], Metformin [255], Thiazolidinediones (pioglitazone) [256], and MSDC-0602K [257]. Various agents such as Aramchol [258,259], Baicalin [260], Nitro-oleic acid [261], Carboxyatractyloside [262], Genistein [263], and Firsocostat (acetyl-CoA carboxylase (ACC) inhibitor) [264]. Mitotherapy implies exogenous mitochondria tagged with green-fluorescence protein (GFP), retrieved in mouse liver, lungs, brain, muscle, and kidneys [265,266]. In this case, the improved energy production may restore hepatocyte function [267]

In conclusion, there is no standard therapy for NAFLD, apart from lifestyle changes helping weight maintenance or weight loss to achieve an ideal body weight. The role of combination therapies to act on different targets simultaneously is being actively investigated.

## 8. Future Perspectives and Conclusions

The interpretation of the results investigating the mitochondrial function by ^13^C- BT in vivo requires some considerations. Studies show that both marked steatosis and NASH, and ethanol consumption, cause mitochondrial dysfunction, which becomes detectable by KICA and methionine BT. This aspect should be particularly useful in the case of early treatments possibly limiting the progression of the disease, and in terms of secondary prevention measures. On the other hand, experimental studies indicate that mitochondrial dysfunction can precede the onset and progression of NAFLD, and these findings also pave the way to possible primary prevention measures. Several factors can influence results from BT, affecting liver perfusion and/or mitochondrial performances. Thus, particular attention is required in the selection of subjects undergoing ^13^C- BT and in confounding factors, also considering possible inter-individual differences. ‘Competing’ mitochondria may be active in extra-hepatic tissues (e.g., muscle). The production of CO_2_ can vary substantially among subjects [268] and therefore the labelled CO_2_ in breath might become independent of circulating or renally excreted bicarbonate and the endogenous production of unlabeled CO_2_. Thus, adequately validated BT will have clinical utility for diagnosis, prognosis, or efficacy of treatments. Remaining limitations of BT applications in clinical practice should be overcome by further translational studies and clinical trials, in parallel with complementary diagnostic techniques. The implementation of novel substrates to investigate additional mitochondrial pathways is greatly warranted.

## Figures and Tables

**Table 1:** General characteristics of an ideal substrate for studying dynamic liver function [143].

**Pharmacokinetic and metabolic aspects**
Rapidly and consistently absorbed by oral route
Primary liver metabolization
Low hepatic extraction ratio (20–30%) (i.e., metabolism independent from liver blood flow)
Clear metabolic pathway Simple pharmacokinetic Short elimination half-life
Minimal compartmentalization of generated ^13^CO_2_ Early appearance of ^13^CO_2_ in breath
**Methodological aspects**
Safe, without side effects
Test simple to prepare and administer
No (or minimal) interaction with extra-hepatic tissues (i.e., adipose tissue, muscle)
Reproducible over time Repeatable (useful for follow-up)
**Costs**
Affordable

**Table 2:** Factors potentially affecting the use of ^13^C-breath tests for the assessment of liver function.

**Increased Total Amount of CO_2_ Production.**
Elderly Increased physical activity Consumed meal Sparkling beverage Respiratory diseases Fever
**Altered liver perfusion**
Anemia Chronic heart failure Transjugular portosystemic shunt
**Altered gastrointestinal function**
Delayed gastric emptying. Altered gastrointestinal absorption.
**Induction of CYP450 1A2**
Chronic cigarette smoking Marijuana Brussel spouts Cabbage Caffeine Carbamezepine Cauliflower Charbroiled foods Clarithromycin Erythromycin Esomeprazole Griseifulvin Insulin Lansoprazole Moricizine Omeprazole Phenobarbital Phenytoin Rifampin Ritonavir
**Inhibition of CYP450 1A2**
Anastrazole Caffeine Cimetidine Ciprofloxacin Enoxacin Fluphenazine Flutamide Fluvoxamine Grapefruit juice Grepafloxacin Isoniazid Lidocaine Lomeflozacin Mexiletine Mibefradil Nelfinavir Norfloxacin Ofloxacin Oral contraceptives Perphenazine Phenacetin Propafenone Ranitidine Rifampin Ropinirole Sparfloxacin Tacrine Ticlopidine Verapamil Zafirlukast

**Table 3:** Liver mitochondrial breath tests: substrates and evidence for potential clinical applications.

Substrate/Clinical Applications	Information
**KICA**	
ALD	Effect of acute alcohol consumption (even low-moderate doses) [171]
ALD	Discrimination between chronic alcohol consumption and nonalcoholic chronic liver disease [173]
ALD	Monitoring and ascertaining of alcohol withdrawal [173]
NAFLD	Discrimination between simple steatosis and steatohepatitis (NASH) and between low-grade and high-grade fibrosis [147] (Figure 6)
HCC	Effect of treatment (thermoablation, chemoembolization) and prediction of tumour recurrence after local treatment. Comparison with methacetin [158]
Drugs	Evaluation of acute drug toxicity [171]
**Methionine**	
Healthy subjects	Validation studies with 2 mg/kg body weight (methyl-^13^C)-methionine. Breath ^13^CO_2_ enrichment measured at base line and every 15 min thereafter for 180 min. [174]
Healthy subjects	Effect of alcohol consumption (30 min after the ingestion of ethanol 0.3 g/kg body weight). Decreased excretion with ethanol, due to impaired mitochondrial oxidation [175].
Liver cirrhosis	Discrimination between different degree of chronic liver damage [175]
ALD	Diagnosis of acute alcohol ingestion [174]
NAFLD	Discrimination between simple steatosis and NASH [176]
HCV	Discrimination between HCV infected patients and healthy subjects and toxicity of pegylated interferon plus ribavirin treatment [177]
Drugs	Evaluation of chronic drug toxicity [178]
Friedriech’s ataxia	Diagnosis of neurological disorders [179]
**Octanoate**	
NAFLD	Evaluation of altered lipid metabolism [180]
NASH	Total β-oxidation of octanoic acid remained normal between controls and NASH patients, although cumulative ^13^CO_2_ recovery was higher in women than men [181]

Abbreviations: ALD = alcoholic liver disease; HCC = hepatocellular carcinoma; HCV = hepatitics C virus; KICA= α-ketoisocaproic acid; NAFLD= nonalcoholic fatty liver disease; NASH = nonalcoholic steatohepatitis.

**Table 4:** Arguments for assessing liver mitochondrial function in NAFLD patients.

Increasing Scientific Interest About the Role of Mitochondria in NAFLD
Impaired liver mitochondrial function may occur early during the onset and progression of NAFLD.
General measures for NAFLD can be beneficial to liver mitochondria as well.
Few medications show some beneficial effects on liver mitochondria.
Improved mitochondrial function can contribute to ameliorate other liver dysfunctions in NAFLD patients.

## References

[1] Lee J., Park J.S., Roh Y.S. (2019). Molecular insights into the role of mitochondria in non-alcoholic fatty liver disease. Arch. Pharm. Res..

[2] Xiang L., Shao Y., Chen Y. (2021). Mitochondrial dysfunction and mitochondrion-targeted therapeutics in liver diseases. J. Drug Target..

[3] Luangmonkong T., Suriguga S., Mutsaers H.A.M., Groothuis G.M.M., Olinga P., Boersema M. (2018). Targeting Oxidative Stress for the Treatment of Liver Fibrosis. Rev. Physiol. Biochem. Pharmacol..

[4] Degli Esposti D., Hamelin J., Bosselut N., Saffroy R., Sebagh M., Pommier A., Martel C., Lemoine A. (2012). Mitochondrial roles and cytoprotection in chronic liver injury. Biochem. Res. Int..

[5] Wei Y., Rector R.S., Thyfault J.P., Ibdah J.A. (2008). Nonalcoholic fatty liver disease and mitochondrial dysfunction. World J. Gastroenterol. WJG.

[6] Guerrieri F., Nicoletti C., Adorisio E., Caraccio G., Leonetti P., Zanotti F., Cantatore P. (2000). Correlation between decreased expression of mitochondrial F0F1-ATP synthase and low regenerating capability of the liver after partial hepatectomy in hypothyroid rats. J. Bioenerg. Biomembr..

[7] Diogo C.V., Grattagliano I., Oliveira P.J., Bonfrate L., Portincasa P. (2011). Re-wiring the circuit: Mitochondria as a pharmacological target in liver disease. Curr. Med. Chem..

[8] Grattagliano I., Russmann S., Diogo C., Bonfrate L., Oliveira P.J., Wang D.Q., Portincasa P. (2011). Mitochondria in chronic liver disease. Curr. Drug. Targets.

[9] Grattagliano I., de Bari O., Bernardo T.C., Oliveira P.J., Wang D.Q., Portincasa P. (2012). Role of mitochondria in nonalcoholic fatty liver disease--from origin to propagation. Clin. Biochem..

[10] Grattagliano I., De Bari O., Di Palo D., Montecucco F., Carbone F., Oliveira P., Wang D.Q.H., Portincasa P., Oliveira P. (2018). Mitochondria in liver diseases. Mitochondrial Biology and Experimental Therapeutics.

[11] Donnelly K.L., Smith C.I., Schwarzenberg S.J., Jessurun J., Boldt M.D., Parks E.J. (2005). Sources of fatty acids stored in liver and secreted via lipoproteins in patients with nonalcoholic fatty liver disease. J. Clin. Investig..

[12] De Angelis M., Garruti G., Minervini F., Bonfrate L., Portincasa P., Gobbetti M. (2019). The Food-gut Human Axis: The Effects of Diet on Gut Microbiota and Metabolome. Curr. Med. Chem..

[13] Di Ciaula A., Garruti G., Lunardi Baccetto R., Molina-Molina E., Bonfrate L., Wang D.Q., Portincasa P. (2017). Bile Acid Physiology. Ann. Hepatol..

[14] Arab J.P., Arrese M., Trauner M. (2018). Recent Insights into the Pathogenesis of Nonalcoholic Fatty Liver Disease. Annu. Rev. Pathol..

[15] Bai L., Li H. (2019). Innate immune regulatory networks in hepatic lipid metabolism. J. Mol. Med..

[16] Di Ciaula A., Passarella S., Shanmugam H., Noviello M., Bonfrate L., Wang D.Q.-H., Portincasa P. (2021). Nonalcoholic Fatty Liver Disease (NAFLD). Mitochondria as Players and Targets of Therapies?. Int. J. Mol. Sci..

[17] Samuel V.T., Shulman G.I. (2018). Nonalcoholic fatty liver disease as a nexus of metabolic and hepatic diseases. Cell metab..

[18] Ono M., Okamoto N., Saibara T. (2010). The latest idea in NAFLD/NASH pathogenesis. Clin. J. Gastroenterol..

[19] Paventi G., Pizzuto R., Passarella S. (2017). The occurrence of l-lactate dehydrogenase in the inner mitochondrial compartment of pig liver. Biochem. Biophys. Res. Commun..

[20] Passarella S., de Bari L., Valenti D., Pizzuto R., Paventi G., Atlante A. (2008). Mitochondria and L-lactate metabolism. FEBS Lett..

[21] Chen Z., Tian R., She Z., Cai J., Li H. (2020). Role of oxidative stress in the pathogenesis of nonalcoholic fatty liver disease. Free Radic. Biol. Med..

[22] European Association for the Study of the Liver (EASL), European Association for the Study of Diabetes (EASD), European Association for the Study of Obesity (EASO) (2016). EASL-EASD-EASO Clinical Practice Guidelines for the management of non-alcoholic fatty liver disease. J. Hepatol..

[23] Cohen J.C., Horton J.D., Hobbs H.H. (2011). Human fatty liver disease: Old questions and new insights. Science.

[24] Szczepaniak L.S., Nurenberg P., Leonard D., Browning J.D., Reingold J.S., Grundy S., Hobbs H.H., Dobbins R.L. (2005). Magnetic resonance spectroscopy to measure hepatic triglyceride content: Prevalence of hepatic steatosis in the general population. Am. J. Physiol. Endocrinol. Metab..

[25] Loomba R., Friedman S.L., Shulman G.I. (2021). Mechanisms and disease consequences of nonalcoholic fatty liver disease. Cell.

[26] Chalasani N., Younossi Z., Lavine J.E., Charlton M., Cusi K., Rinella M., Harrison S.A., Brunt E.M., Sanyal A.J. (2018). The diagnosis and management of nonalcoholic fatty liver disease: Practice guidance from the American Association for the Study of Liver Diseases. Hepatology.

[27] Singh S., Allen A.M., Wang Z., Prokop L.J., Murad M.H., Loomba R. (2015). Fibrosis progression in nonalcoholic fatty liver vs nonalcoholic steatohepatitis: A systematic review and meta-analysis of paired-biopsy studies. Clin. Gastroenterol. Hepatol..

[28] Ludwig J., Viggiano T.R., McGill D.B., Oh B.J. (1980). Nonalcoholic steatohepatitis: Mayo Clinic experiences with a hitherto unnamed disease. Mayo Clin. Proc. Mayo Clin..

[29] Caldwell S.H., Oelsner D.H., Iezzoni J.C., Hespenheide E.E., Battle E.H., Driscoll C.J. (1999). Cryptogenic cirrhosis: Clinical characterization and risk factors for underlying disease. Hepatology.

[30] Browning J.D., Kumar K.S., Saboorian M.H., Thiele D.L. (2004). Ethnic differences in the prevalence of cryptogenic cirrhosis. Am. J. Gastroenterol..

[31] Nasr P., Ignatova S., Kechagias S., Ekstedt M. (2018). Natural history of nonalcoholic fatty liver disease: A prospective follow-up study with serial biopsies. Hepatol. Commun..

[32] Younossi Z., Anstee Q.M., Marietti M., Hardy T., Henry L., Eslam M., George J., Bugianesi E. (2018). Global burden of NAFLD and NASH: Trends, predictions, risk factors and prevention. Nat. Rev. Gastroenterol. Hepatol..

[33] Mittal S., El-Serag H.B., Sada Y.H., Kanwal F., Duan Z., Temple S., May S.B., Kramer J.R., Richardson P.A., Davila J.A. (2016). Hepatocellular Carcinoma in the Absence of Cirrhosis in United States Veterans is Associated With Nonalcoholic Fatty Liver Disease. Clin. Gastroenterol. Hepatol..

[34] Dulai P.S., Singh S., Patel J., Soni M., Prokop L.J., Younossi Z., Sebastiani G., Ekstedt M., Hagstrom H., Nasr P. (2017). Increased risk of mortality by fibrosis stage in nonalcoholic fatty liver disease: Systematic review and meta-analysis. Hepatology.

[35] Eslam M., Newsome P.N., Sarin S.K., Anstee Q.M., Targher G., Romero-Gomez M., Zelber-Sagi S., Wai-Sun Wong V., Dufour J.F., Schattenberg J.M. (2020). A new definition for metabolic dysfunction-associated fatty liver disease: An international expert consensus statement. J. Hepatol..

[36] Younossi Z.M., Rinella M.E., Sanyal A.J., Harrison S.A., Brunt E.M., Goodman Z., Cohen D.E., Loomba R. (2021). From NAFLD to MAFLD: Implications of a Premature Change in Terminology. Hepatology.

[37] Di Ciaula A., Baj J., Garruti G., Celano G., De Angelis M., Wang H.H., Di Palo D.M., Bonfrate L., Wang D.Q.-H., Portincasa P. (2020). Liver Steatosis, Gut-Liver Axis, Microbiome and Environmental Factors. A Never-Ending Bidirectional Cross-Talk. J. Clin. Med..

[38] Di Palo D.M., Garruti G., Di Ciaula A., Molina-Molina E., Shanmugam H., De Angelis M., Portincasa P. (2020). Increased Colonic Permeability and Lifestyles as Contributing Factors to Obesity and Liver Steatosis. Nutrients.

[39] Di Ciaula A., Carbone F., Shanmugham H., Molina-Molina E., Bonfrate L., Ministrini S., Montecucco F., Portincasa P. (2021). Adiponectin involved in portal flow hepatic extraction of 13C-metacethin in obesity and non-alcoholic fatty liver. Eur. J. Intern. Med..

[40] Williams C.D., Stengel J., Asike M.I., Torres D.M., Shaw J., Contreras M., Landt C.L., Harrison S.A. (2011). Prevalence of nonalcoholic fatty liver disease and nonalcoholic steatohepatitis among a largely middle-aged population utilizing ultrasound and liver biopsy: A prospective study. Gastroenterology.

[41] Vernon G., Baranova A., Younossi Z.M. (2011). Systematic review: The epidemiology and natural history of non-alcoholic fatty liver disease and non-alcoholic steatohepatitis in adults. Aliment. Pharmacol. Ther..

[42] Lazo M., Hernaez R., Eberhardt M.S., Bonekamp S., Kamel I., Guallar E., Koteish A., Brancati F.L., Clark J.M. (2013). Prevalence of nonalcoholic fatty liver disease in the United States: The Third National Health and Nutrition Examination Survey, 1988–1994. Am. J. Epidemiol..

[43] Younossi Z.M., Stepanova M., Afendy M., Fang Y., Younossi Y., Mir H., Srishord M. (2011). Changes in the prevalence of the most common causes of chronic liver diseases in the United States from 1988 to 2008. Clin. Gastroenterol. Hepatol..

[44] Younossi Z., Tacke F., Arrese M., Chander Sharma B., Mostafa I., Bugianesi E., Wai-Sun Wong V., Yilmaz Y., George J., Fan J. (2019). Global Perspectives on Nonalcoholic Fatty Liver Disease and Nonalcoholic Steatohepatitis. Hepatology.

[45] Molina-Molina E., Lunardi Baccetto R., Wang D.Q., de Bari O., Krawczyk M., Portincasa P. (2018). Exercising the hepatobiliary-gut axis. The impact of physical activity performance. Eur. J. Clin. Investig..

[46] Molina-Molina E., Krawczyk M., Stachowska E., Lammert F., Portincasa P. (2019). Non-Alcoholic Fatty Liver Disease in Non-Obese Individuals: Prevalence, Pathogenesis and Treatment. Clin. Res. Hepatol. Gastroenterol..

[47] Zhou J., Zhou F., Wang W., Zhang X.J., Ji Y.X., Zhang P., She Z.G., Zhu L., Cai J., Li H. (2020). Epidemiological Features of NAFLD From 1999 to 2018 in China. Hepatology.

[48] Kim D., Kim W.R. (2017). Nonobese Fatty Liver Disease. Clin. Gastroenterol. Hepatol..

[49] Yoshitaka H., Hamaguchi M., Kojima T., Fukuda T., Ohbora A., Fukui M. (2017). Nonoverweight nonalcoholic fatty liver disease and incident cardiovascular disease: A post hoc analysis of a cohort study. Medicine.

[50] Adams L.A., Lymp J.F., St Sauver J., Sanderson S.O., Lindor K.D., Feldstein A., Angulo P. (2005). The natural history of nonalcoholic fatty liver disease: A population-based cohort study. Gastroenterology.

[51] Lindenmeyer C.C., McCullough A.J. (2018). The Natural History of Nonalcoholic Fatty Liver Disease-An Evolving View. Clin. Liver Dis..

[52] Rinella M.E., Sanyal A.J. (2016). Management of NAFLD: A stage-based approach. Nat. Rev. Gastroenterol. Hepatol..

[53] Caussy C., Soni M., Cui J., Bettencourt R., Schork N., Chen C.H., Ikhwan M.A., Bassirian S., Cepin S., Gonzalez M.P. (2017). Nonalcoholic fatty liver disease with cirrhosis increases familial risk for advanced fibrosis. J. Clin. Investig..

[54] Stender S., Loomba R. (2020). PNPLA3 Genotype and Risk of Liver and All-Cause Mortality. Hepatology.

[55] Krawczyk M., Portincasa P., Lammert F. (2013). PNPLA3-associated steatohepatitis: Toward a gene-based classification of fatty liver disease. Semin. Liver Dis..

[56] Loomba R., Lim J.K., Patton H., El-Serag H.B. (2020). AGA Clinical Practice Update on Screening and Surveillance for Hepatocellular Carcinoma in Patients With Nonalcoholic Fatty Liver Disease: Expert Review. Gastroenterology.

[57] Portincasa P., Wang D.Q.H., Podolsky K.D., Camilleri M., Fitz J.G., Kalloo A.N., Shanahan F., Wang T.C. (2015). Gallstones. Yamada’s Textbook of Gastroenterology.

[58] Portincasa P., Moschetta A., Palasciano G. (2006). Cholesterol gallstone disease. Lancet.

[59] National Institute of Alcohol Abuse and Alcoholism (NIH). https://pubs.niaaa.nih.gov/publications/practitioner/pocketguide/pocket_guide2.htm.

[60] Torres D.M., Williams C.D., Harrison S.A. (2012). Features, diagnosis, and treatment of nonalcoholic fatty liver disease. Clin. Gastroenterol. Hepatol..

[61] Sherlock S., Dooley J. (2002). Diseases of the Liver and Biliary System.

[62] Palmentieri B., de Sio I., La Mura V., Masarone M., Vecchione R., Bruno S., Torella R., Persico M. (2006). The role of bright liver echo pattern on ultrasound B-mode examination in the diagnosis of liver steatosis. Dig. Liver Dis..

[63] Park Y.S., Park S.H., Lee S.S., Kim D.Y., Shin Y.M., Lee W., Lee S.G., Yu E.S. (2011). Biopsy-proven nonsteatotic liver in adults: Estimation of reference range for difference in attenuation between the liver and the spleen at nonenhanced CT. Radiology.

[64] Wells M.M., Li Z., Addeman B., McKenzie C.A., Mujoomdar A., Beaton M., Bird J. (2016). Computed Tomography Measurement of Hepatic Steatosis: Prevalence of Hepatic Steatosis in a Canadian Population. Can. J. Gastroenterol. Hepatol..

[65] Bohte A.E., van Werven J.R., Bipat S., Stoker J. (2011). The diagnostic accuracy of US, CT, MRI and 1H-MRS for the evaluation of hepatic steatosis compared with liver biopsy: A meta-analysis. Eur. Radiol..

[66] Zhang Y.N., Fowler K.J., Hamilton G., Cui J.Y., Sy E.Z., Balanay M., Hooker J.C., Szeverenyi N., Sirlin C.B. (2018). Liver fat imaging-a clinical overview of ultrasound, CT, and MR imaging. Br. J. Radiol..

[67] Shanmugam H., Molina Molina E., Di Palo D.M., Faienza M.F., Di Ciaula A., Garruti G., Wang D.Q.H., Portincasa P. (2020). Physical Activity Modulating Lipid Metabolism in Gallbladder Diseases. J. Gastrointest. Liver Dis..

[68] Caldwell S.H., Swerdlow R.H., Khan E.M., Iezzoni J.C., Hespenheide E.E., Parks J.K., Parker W.D. (1999). Mitochondrial abnormalities in non-alcoholic steatohepatitis. J. Hepatol..

[69] Simoes I.C.M., Karkucinska-Wieckowska A., Janikiewicz J., Szymanska S., Pronicki M., Dobrzyn P., Dabrowski M., Dobrzyn A., Oliveira P.J., Zischka H. (2020). Western Diet Causes Obesity-Induced Nonalcoholic Fatty Liver Disease Development by Differentially Compromising the Autophagic Response. Antioxidants.

[70] Longo M., Meroni M., Paolini E., Macchi C., Dongiovanni P. (2021). Mitochondrial dynamics and nonalcoholic fatty liver disease (NAFLD): New perspectives for a fairy-tale ending?. Metab. Clin. Exp..

[71] Ajaz S., McPhail M.J., Gnudi L., Trovato F.M., Mujib S., Napoli S., Carey I., Agarwal K. (2021). Mitochondrial dysfunction as a mechanistic biomarker in patients with non-alcoholic fatty liver disease (NAFLD). Mitochondrion.

[72] Shannon C.E., Ragavan M., Palavicini J.P., Fourcaudot M., Bakewell T.M., Valdez I.A., Ayala I., Jin E.S., Madesh M., Han X. (2021). Insulin resistance is mechanistically linked to hepatic mitochondrial remodeling in non-alcoholic fatty liver disease. Mol. Metab..

[73] Li Y., Wu J., Yang M., Wei L., Wu H., Wang Q., Shi H. (2021). Physiological evidence of mitochondrial permeability transition pore opening caused by lipid deposition leading to hepatic steatosis in db/db mice. Free Radic. Biol. Med..

[74] Yan C., Duanmu X., Zeng L., Liu B., Song Z. (2019). Mitochondrial DNA: Distribution, Mutations, and Elimination. Cells.

[75] Garcia-Martinez I., Santoro N., Chen Y., Hoque R., Ouyang X., Caprio S., Shlomchik M.J., Coffman R.L., Candia A., Mehal W.Z. (2016). Hepatocyte mitochondrial DNA drives nonalcoholic steatohepatitis by activation of TLR9. J. Clin. Investig..

[76] Pan J., Ou Z., Cai C., Li P., Gong J., Ruan X.Z., He K. (2018). Fatty acid activates NLRP3 inflammasomes in mouse Kupffer cells through mitochondrial DNA release. Cell Immunol..

[77] Pirola C.J., Garaycoechea M., Flichman D., Castano G.O., Sookoian S. (2021). Liver mitochondrial DNA damage and genetic variability of Cytochrome b—A key component of the respirasome drive the severity of fatty liver disease. J. Intern. Med..

[78] Malik A.N., Czajka A. (2013). Is mitochondrial DNA content a potential biomarker of mitochondrial dysfunction?. Mitochondrion.

[79] Begriche K., Massart J., Robin M.A., Bonnet F., Fromenty B. (2013). Mitochondrial adaptations and dysfunctions in nonalcoholic fatty liver disease. Hepatology.

[80] Masarone M., Rosato V., Dallio M., Gravina A.G., Aglitti A., Loguercio C., Federico A., Persico M. (2018). Role of Oxidative Stress in Pathophysiology of Nonalcoholic Fatty Liver Disease. Oxidative Med. Cell. Longev..

[81] Seki S., Kitada T., Yamada T., Sakaguchi H., Nakatani K., Wakasa K. (2002). In situ detection of lipid peroxidation and oxidative DNA damage in non-alcoholic fatty liver diseases. J. Hepatol..

[82] Weltman M.D., Farrell G.C., Liddle C. (1996). Increased hepatocyte CYP2E1 expression in a rat nutritional model of hepatic steatosis with inflammation. Gastroenterology.

[83] Chalasani N., Gorski J.C., Asghar M.S., Asghar A., Foresman B., Hall S.D., Crabb D.W. (2003). Hepatic cytochrome P450 2E1 activity in nondiabetic patients with nonalcoholic steatohepatitis. Hepatology.

[84] Aubert J., Begriche K., Knockaert L., Robin M.A., Fromenty B. (2011). Increased expression of cytochrome P450 2E1 in nonalcoholic fatty liver disease: Mechanisms and pathophysiological role. Clin. Res. Hepatol. Gastroenterol..

[85] Caballero F., Fernandez A., Matias N., Martinez L., Fucho R., Elena M., Caballeria J., Morales A., Fernandez-Checa J.C., Garcia-Ruiz C. (2010). Specific contribution of methionine and choline in nutritional nonalcoholic steatohepatitis: Impact on mitochondrial S-adenosyl-L-methionine and glutathione. J. Biol. Chem..

[86] Gao D., Wei C., Chen L., Huang J., Yang S., Diehl A.M. (2004). Oxidative DNA damage and DNA repair enzyme expression are inversely related in murine models of fatty liver disease. Am. J. Physiol. Gastrointest. Liver Physiol..

[87] Tesse A., Grossini E., Tamma G., Brenner C., Portincasa P., Marinelli R.A., Calamita G. (2018). Aquaporins as targets of dietary bioactive phytocompounds. Front. Mol. Biosci..

[88] Calamita G., Portincasa P. (2016). The power of science diplomacy, a lesson from the Nobel laureate Peter Agre. Eur. J. Clin. Investig..

[89] Portincasa P., Palasciano G., Svelto M., Calamita G. (2008). Aquaporins in the hepatobiliary tract. Which, where and what they do in health and disease. Eur. J. Clin. Investig..

[90] Ferri D., Mazzone A., Liquori G.E., Cassano G., Svelto M., Calamita G. (2003). Ontogeny, distribution, and possible functional implications of an unusual aquaporin, AQP8, in mouse liver. Hepatology.

[91] Marchissio M.J., Frances D.E., Carnovale C.E., Marinelli R.A. (2012). Mitochondrial aquaporin-8 knockdown in human hepatoma HepG2 cells causes ROS-induced mitochondrial depolarization and loss of viability. Toxicol. Appl. Pharmacol..

[92] Busanello E.N.B., Figueira T.R., Marques A.C., Navarro C.D.C., Oliveira H.C.F., Vercesi A.E. (2018). Facilitation of Ca(2+) -induced opening of the mitochondrial permeability transition pore either by nicotinamide nucleotide transhydrogenase deficiency or statins treatment. Cell Biol. Int..

[93] Slater T.F. (1988). Free radical mechanisms in tissue injury. Cell Funct. Dis..

[94] Petrosillo G., Portincasa P., Grattagliano I., Casanova G., Matera M., Ruggiero F.M., Ferri D., Paradies G. (2007). Mitochondrial dysfunction in rat with nonalcoholic fatty liver Involvement of complex I, reactive oxygen species and cardiolipin. Biochim. Biophys. Acta.

[95] Grattagliano I., Caraceni P., Calamita G., Ferri D., Gargano I., Palasciano G., Portincasa P. (2008). Severe liver steatosis correlates with nitrosative and oxidative stress in rats. Eur. J. Clin. Investig..

[96] Grattagliano I., Lauterburg B.H., Palasciano G., Portincasa P. (2010). 13C-breath tests for clinical investigation of liver mitochondrial function. Eur. J. Clin. Investig..

[97] Sunny N.E., Parks E.J., Browning J.D., Burgess S.C. (2011). Excessive hepatic mitochondrial TCA cycle and gluconeogenesis in humans with nonalcoholic fatty liver disease. Cell Metab..

[98] Schmid A.I., Szendroedi J., Chmelik M., Krssak M., Moser E., Roden M. (2011). Liver ATP synthesis is lower and relates to insulin sensitivity in patients with type 2 diabetes. Diabetes Care.

[99] Peng K.Y., Watt M.J., Rensen S., Greve J.W., Huynh K., Jayawardana K.S., Meikle P.J., Meex R.C.R. (2018). Mitochondrial dysfunction-related lipid changes occur in nonalcoholic fatty liver disease progression. J. Lipid Res..

[100] Koliaki C., Szendroedi J., Kaul K., Jelenik T., Nowotny P., Jankowiak F., Herder C., Carstensen M., Krausch M., Knoefel W.T. (2015). Adaptation of hepatic mitochondrial function in humans with non-alcoholic fatty liver is lost in steatohepatitis. Cell Metab..

[101] Fletcher J.A., Deja S., Satapati S., Fu X., Burgess S.C., Browning J.D. (2019). Impaired ketogenesis and increased acetyl-CoA oxidation promote hyperglycemia in human fatty liver. JCI Insight.

[102] Grasselli E., Baldini F., Vecchione G., Oliveira P.J., Sardao V.A., Voci A., Portincasa P., Vergani L. (2019). Excess fructose and fatty acids trigger a model of nonalcoholic fatty liver disease progression in vitro: Protective effect of the flavonoid silybin. Int. J. Mol. Med..

[103] An P., Wei L.L., Zhao S., Sverdlov D.Y., Vaid K.A., Miyamoto M., Kuramitsu K., Lai M., Popov Y.V. (2020). Hepatocyte mitochondria-derived danger signals directly activate hepatic stellate cells and drive progression of liver fibrosis. Nat. Commun..

[104] Grossini E., Garhwal D.P., Calamita G., Romito R., Rigamonti C., Minisini R., Smirne C., Surico D., Bellan M., Pirisi M. (2021). Exposure to Plasma From Non-alcoholic Fatty Liver Disease Patients Affects Hepatocyte Viability, Generates Mitochondrial Dysfunction, and Modulates Pathways Involved in Fat Accumulation and Inflammation. Front. Med..

[105] Reddy J.K. (2001). Nonalcoholic steatosis and steatohepatitis. III. Peroxisomal beta-oxidation, PPAR alpha, and steatohepatitis. Am. J. Physiol. Gastrointest. Liver Physiol..

[106] Camporez J.P., Wang Y., Faarkrog K., Chukijrungroat N., Petersen K.F., Shulman G.I. (2017). Mechanism by which arylamine *N*-acetyltransferase 1 ablation causes insulin resistance in mice. Proc. Natl. Acad. Sci. USA.

[107] Chennamsetty I., Coronado M., Contrepois K., Keller M.P., Carcamo-Orive I., Sandin J., Fajardo G., Whittle A.J., Fathzadeh M., Snyder M. (2016). Nat1 Deficiency Is Associated with Mitochondrial Dysfunction and Exercise Intolerance in Mice. Cell Rep..

[108] Gijón M.A., Riekhof W.R., Zarini S., Murphy R.C., Voelker D.R. (2008). Lysophospholipid Acyltransferases and Arachidonate Recycling in Human Neutrophils*. J. Biol. Chem..

[109] Mancina R.M., Dongiovanni P., Petta S., Pingitore P., Meroni M., Rametta R., Boren J., Montalcini T., Pujia A., Wiklund O. (2016). The MBOAT7-TMC4 Variant rs641738 Increases Risk of Nonalcoholic Fatty Liver Disease in Individuals of European Descent. Gastroenterology.

[110] Luukkonen P.K., Zhou Y., Hyötyläinen T., Leivonen M., Arola J., Orho-Melander M., Orešič M., Yki-Järvinen H. (2016). The MBOAT7 variant rs641738 alters hepatic phosphatidylinositols and increases severity of non-alcoholic fatty liver disease in humans. J. Hepatol..

[111] Buch S., Stickel F., Trépo E., Way M., Herrmann A., Nischalke H.D., Brosch M., Rosendahl J., Berg T., Ridinger M. (2015). A genome-wide association study confirms PNPLA3 and identifies TM6SF2 and MBOAT7 as risk loci for alcohol-related cirrhosis. Nat. Genet..

[112] Thabet K., Asimakopoulos A., Shojaei M., Romero-Gomez M., Mangia A., Irving W.L., Berg T., Dore G.J., Grønbæk H., Sheridan D. (2016). MBOAT7 rs641738 increases risk of liver inflammation and transition to fibrosis in chronic hepatitis C. Nat. Commun..

[113] Thangapandi V.R., Knittelfelder O., Brosch M., Patsenker E., Vvedenskaya O., Buch S., Hinz S., Hendricks A., Nati M., Herrmann A. (2021). Loss of hepatic Mboat7 leads to liver fibrosis. Gut.

[114] Helsley R.N., Venkateshwari V., Brown A.L., Gromovsky A.D., Schugar R.C., Ramachandiran I., Fung K., Kabbany M.N., Banerjee R., Neumann C.K. (2019). Obesity-linked suppression of membrane-bound Oacyltransferase 7 (MBOAT7) drives non-alcoholic fatty liver disease. Elife.

[115] Fondevila M.F., Fernandez U., Gonzalez-Rellan M.J., Da Silva Lima N., Buque X., Gonzalez-Rodriguez A., Alonso C., Iruarrizaga-Lejarreta M., Delgado T.C., Varela-Rey M. (2021). The L-α-Lysophosphatidylinositol/G Protein–Coupled Receptor 55 System Induces the Development of Nonalcoholic Steatosis and Steatohepatitis. Hepatology.

[116] Petersen K.F., Befroy D., Dufour S., Dziura J., Ariyan C., Rothman D.L., DiPietro L., Cline G.W., Shulman G.I. (2003). Mitochondrial dysfunction in the elderly: Possible role in insulin resistance. Science.

[117] Lee H.-Y., Choi C.S., Birkenfeld A.L., Alves T.C., Jornayvaz F.R., Jurczak M.J., Zhang D., Woo D.K., Shadel G.S., Ladiges W. (2010). Targeted Expression of Catalase to Mitochondria Prevents Age-Associated Reductions in Mitochondrial Function and Insulin Resistance. Cell Metab..

[118] Palmer A.K., Kirkland J.L. (2016). Aging and adipose tissue: Potential interventions for diabetes and regenerative medicine. Exp. Gerontol..

[119] Lyu K., Zhang Y., Zhang D., Kahn M., ter Horst K.W., Rodrigues M.R.S., Gaspar R.C., Hirabara S.M., Luukkonen P.K., Lee S. (2020). A Membrane-Bound Diacylglycerol Species Induces PKCϵ-Mediated Hepatic Insulin Resistance. Cell Metab..

[120] Piccinin E., Villani G., Moschetta A. (2019). Metabolic aspects in NAFLD, NASH and hepatocellular carcinoma: The role of PGC1 coactivators. Nat. Rev. Gastroenterol. Hepatol..

[121] Li Y., Chen C., Lu L., Guo W., VanWagner L.B., Shikany J.M., Zhang S., Kahe K. (2021). Cadmium Exposure in Young Adulthood Is Associated with Risk of Nonalcoholic Fatty Liver Disease in Midlife. Dig. Dis. Sci..

[122] He X., Gao J., Hou H., Qi Z., Chen H., Zhang X.X. (2019). Inhibition of Mitochondrial Fatty Acid Oxidation Contributes to Development of Nonalcoholic Fatty Liver Disease Induced by Environmental Cadmium Exposure. Environ. Sci. Technol..

[123] Frediani J.K., Naioti E.A., Vos M.B., Figueroa J., Marsit C.J., Welsh J.A. (2018). Arsenic exposure and risk of nonalcoholic fatty liver disease (NAFLD) among U.S. adolescents and adults: An association modified by race/ethnicity, NHANES 2005–2014. Environ. Health Glob. Access Sci. Source.

[124] Mozaffarian F., Dehghani M.A., Vanani A.R., Mahdavinia M. (2021). Protective Effects of Alpha Lipoic Acid Against Arsenic Induced Oxidative Stress in Isolated Rat Liver Mitochondria. Biol. Trace Elem. Res..

[125] Chen R., Xu Y., Xu C., Shu Y., Ma S., Lu C., Mo X. (2019). Associations between mercury exposure and the risk of nonalcoholic fatty liver disease (NAFLD) in US adolescents. Environ. Sci. Pollut. Res. Int..

[126] Pereira L.C., de Paula E.S., Pazin M., Carneiro M.F.H., Grotto D., Barbosa F., Dorta D.J. (2020). Niacin prevents mitochondrial oxidative stress caused by sub-chronic exposure to methylmercury. Drug Chem. Toxicol..

[127] Qiu Y.N., Wang G.H., Zhou F., Hao J.J., Tian L., Guan L.F., Geng X.K., Ding Y.C., Wu H.W., Zhang K.Z. (2019). PM2.5 induces liver fibrosis via triggering ROS-mediated mitophagy. Ecotoxicol. Environ. Saf..

[128] Breton C.V., Song A.Y., Xiao J., Kim S.J., Mehta H.H., Wan J., Yen K., Sioutas C., Lurmann F., Xue S. (2019). Effects of air pollution on mitochondrial function, mitochondrial DNA methylation, and mitochondrial peptide expression. Mitochondrion.

[129] Chen J., Wu L., Yang G., Zhang C., Liu X., Sun X., Chen X., Wang N. (2021). The influence of PM2.5 exposure on non-alcoholic fatty liver disease. Life Sci..

[130] Khan S., Beigh S., Chaudhari B.P., Sharma S., Aliul Hasan Abdi S., Ahmad S., Ahmad F., Parvez S., Raisuddin S. (2016). Mitochondrial dysfunction induced by Bisphenol A is a factor of its hepatotoxicity in rats. Environ. Toxicol..

[131] Huc L., Lemarie A., Gueraud F., Helies-Toussaint C. (2012). Low concentrations of bisphenol A induce lipid accumulation mediated by the production of reactive oxygen species in the mitochondria of HepG2 cells. Toxicol. Vitr. Int. J. Publ. Assoc. BIBRA.

[132] Liu Q., Wang Q., Xu C., Shao W., Zhang C., Liu H., Jiang Z., Gu A. (2017). Organochloride pesticides impaired mitochondrial function in hepatocytes and aggravated disorders of fatty acid metabolism. Sci. Rep..

[133] Wahlang B., Appana S., Falkner K.C., McClain C.J., Brock G., Cave M.C. (2020). Insecticide and metal exposures are associated with a surrogate biomarker for non-alcoholic fatty liver disease in the National Health and Nutrition Examination Survey 2003-2004. Environ. Sci. Pollut. Res. Int..

[134] Miranda C.A., Guimaraes A., Bizerra P.F.V., Mingatto F.E. (2020). Diazinon impairs bioenergetics and induces membrane permeability transition on mitochondria isolated from rat liver. J. Toxicol. Environ. Health A.

[135] Rives C., Fougerat A., Ellero-Simatos S., Loiseau N., Guillou H., Gamet-Payrastre L., Wahli W. (2020). Oxidative Stress in NAFLD: Role of Nutrients and Food Contaminants. Biomolecules.

[136] Vecchione G., Grasselli E., Voci A., Baldini F., Grattagliano I., Wang D.Q., Portincasa P., Vergani L. (2016). Silybin counteracts lipid excess and oxidative stress in cultured steatotic hepatic cells. World J. Gastroenterol. WJG.

[137] Nadanaciva S., Will Y. (2011). New insights in drug-induced mitochondrial toxicity. Curr. Pharm. Des..

[138] Pereira C.V., Nadanaciva S., Oliveira P.J., Will Y. (2012). The contribution of oxidative stress to drug-induced organ toxicity and its detection in vitro and in vivo. Expert Opin. Drug Metab. Toxicol..

[139] Masuo Y., Imai T., Shibato J., Hirano M., Jones O.A., Maguire M.L., Satoh K., Kikuchi S., Rakwal R. (2009). Omic analyses unravels global molecular changes in the brain and liver of a rat model for chronic Sake (Japanese alcoholic beverage) intake. Electrophoresis.

[140] Griffin J.L., Nicholls A.W. (2006). Metabolomics as a functional genomic tool for understanding lipid dysfunction in diabetes, obesity and related disorders. Pharmacogenomics.

[141] Malik A.N., Simoes I.C.M., Rosa H.S., Khan S., Karkucinska-Wieckowska A., Wieckowski M.R. (2019). A Diet Induced Maladaptive Increase in Hepatic Mitochondrial DNA Precedes OXPHOS Defects and May Contribute to Non-Alcoholic Fatty Liver Disease. Cells.

[142] Molina-Molina E., Shanmugam H., Di Ciaula A., Grattagliano I., Di Palo D.M., Palmieri V.O., Portincasa P. (2021). ((13)C)-Methacetin breath test provides evidence of subclinical liver dysfunction linked to fat storage but not lifestyle. JHEP Rep..

[143] Bonfrate L., Grattagliano I., Palasciano G., Portincasa P. (2015). Dynamic carbon 13 breath tests for the study of liver function and gastric emptying. Gastroenterol. Rep..

[144] Grattagliano I., Bonfrate L., Lorusso M., Castorani L., de Bari O., Portincasa P. (2015). Exploring liver mitochondrial function by (1)(3)C-stable isotope breath tests: Implications in clinical biochemistry. Methods Mol. Biol..

[145] Grattagliano I., Bonfrate L., Oliveira P.J., Castorani L., Ruggiero V., Valenzano A.T., Ascensao A., Buzoianu A., Portincasa P. (2013). Breath tests with novel 13C-substrates for clinical studies of liver mitochondrial function in health and disease. Eur. Rev. Med Pharmacol. Sci..

[146] Perri F., Bellini M., Portincasa P., Parodi A., Bonazzi P., Marzio L., Galeazzi F., Usai P., Citrino A., Usai-Satta P. (2010). (13)C-octanoic acid breath test (OBT) with a new test meal (EXPIROGer): Toward standardization for testing gastric emptying of solids. Dig. Liver Dis..

[147] Portincasa P., Grattagliano I., Lauterburg B.H., Palmieri V.O., Palasciano G., Stellaard F. (2006). Liver breath tests non-invasively predict higher stages of non-alcoholic steatohepatitis. Clin. Sci..

[148] Festi D., Capodicasa S., Sandri L., Colaiocco-Ferrante L., Staniscia T., Vitacolonna E., Vestito A., Simoni P., Mazzella G., Portincasa P. (2005). Measurement of hepatic functional mass by means of 13C-methacetin and 13C-phenylalanine breath tests in chronic liver disease: Comparison with Child-Pugh score and serum bile acid levels. World J. Gastroenterol. WJG.

[149] Portincasa P., Moschetta A., Palasciano G. (2001). Nuovi breath test per lo studio dello svuotamento gastrico e del transito intestinale. Rilevanza in Pazienti Con Stipsi Funzionale.

[150] Gasbarrini A., Corazza G.R., Gasbarrini G., Montalto M., Di Stefano M., Basilisco G., Parodi A., Usai-Satta P., Vernia P., Anania C. (2009). Methodology and indications of H2-breath testing in gastrointestinal diseases: The Rome Consensus Conference. Aliment. Pharmacol. Ther..

[151] Vitellio P., Celano G., Bonfrate L., Gobbetti M., Portincasa P., De Angelis M. (2019). Effects of Bifidobacterium longum and Lactobacillus rhamnosus on Gut Microbiota in Patients with Lactose Intolerance and Persisting Functional Gastrointestinal Symptoms: A Randomised, Double-Blind, Cross-Over Study. Nutrients.

[152] Portincasa P., Di Ciaula A., Vacca M., Montelli R., Wang D.Q., Palasciano G. (2008). Beneficial effects of oral tilactase on patients with hypolactasia. Eur. J. Clin. Investig..

[153] Krawczyk M., Wolska M., Schwartz S., Gruenhage F., Terjung B., Portincasa P., Sauerbruch T., Lammert F. (2008). Concordance of genetic and breath tests for lactose intolerance in a tertiary referral centre. J. Gastrointest. Liver Dis. JGLD.

[154] Bonfrate L., Krawczyk M., Lembo A., Grattagliano I., Lammert F., Portincasa P. (2015). Effects of dietary education, followed by a tailored fructose-restricted diet in adults with fructose malabsorption. Eur. J. Gastroenterol. Hepatol..

[155] Merkel C., Bolognesi M., Bellon S., Bianco S., Honisch B., Lampe H., Angeli P., Gatta A. (1992). Aminopyrine breath test in the prognostic evaluation of patients with cirrhosis. Gut.

[156] Armuzzi A., Candelli M., Zocco M.A., Andreoli A., De Lorenzo A., Nista E.C., Miele L., Cremonini F., Cazzato I.A., Grieco A. (2002). Review article: Breath testing for human liver function assessment. Aliment. Pharmacol. Ther..

[157] Michaletz P.A., Cap L., Alpert E., Lauterburg B.H. (1989). Assessment of mitochondrial function in vivo with a breath test utilizing alpha-ketoisocaproic acid. Hepatology.

[158] Palmieri V.O., Grattagliano I., Minerva F., Pollice S., Palasciano G., Portincasa P. (2009). Liver function as assessed by breath tests in patients with hepatocellular carcinoma. J. Surg. Res..

[159] Dawson B., Trapp R.G. (2001). Basic & Clinical Biostatistics.

[160] Hintze J. (2020). NCSS 2020 Statistical Software.

[161] Gorowska-Kowolik K., Chobot A., Kwiecien J. (2017). (13)C Methacetin Breath Test for Assessment of Microsomal Liver Function: Methodology and Clinical Application. Gastroenterol. Res. Pract..

[162] Afolabi P., Wright M., Wootton S.A., Jackson A.A. (2013). Clinical utility of 13C-liver-function breath tests for assessment of hepatic function. Dig. Dis. Sci..

[163] Stockmann M., Lock J.F., Riecke B., Heyne K., Martus P., Fricke M., Lehmann S., Niehues S.M., Schwabe M., Lemke A.J. (2009). Prediction of postoperative outcome after hepatectomy with a new bedside test for maximal liver function capacity. Ann. Surg..

[164] Buechter M., Kersting S., Gerken G., Kahraman A. (2019). Enzymatic liver function measured by LiMAx—A reliable diagnostic and prognostic tool in chronic liver disease. Sci. Rep..

[165] Holzhutter H.G., Wuensch T., Gajowski R., Berndt N., Bulik S., Meierhofer D., Stockmann M. (2020). A novel variant of the (13)C-methacetin liver function breath test that eliminates the confounding effect of individual differences in systemic CO2 kinetics. Arch. Toxicol..

[166] Schmitz S.M., Kroh A., Ulmer T.F., Andruszkow J., Luedde T., Brozat J.F., Neumann U.P., Alizai P.H. (2020). Evaluation of NAFLD and fibrosis in obese patients—A comparison of histological and clinical scoring systems. BMC Gastroenterol..

[167] Afolabi P.R., Scorletti E., Smith D.E., Almehmadi A.A., Calder P.C., Byrne C.D. (2018). The characterisation of hepatic mitochondrial function in patients with non-alcoholic fatty liver disease (NAFLD) using the 13C-ketoisocaproate breath test. J. Breath Res..

[168] Krahenbuhl L., Ledermann M., Lang C., Krahenbuhl S. (2000). Relationship between hepatic mitochondrial functions in vivo and in vitro in rats with carbon tetrachloride-induced liver cirrhosis. J. Hepatol..

[169] Miele L., Marrone G., Cefalo C., D’Achille S., Rapaccini G.L., Gasbarrini A., Grieco A. (2013). Potential use of liver function breath tests in the clinical practice. Eur. Rev. Med Pharmacol. Sci..

[170] Giannini E., Fasoli A., Chiarbonello B., Malfatti F., Romagnoli P., Botta F., Testa E., Polegato S., Fumagalli A., Testa R. (2002). 13C-aminopyrine breath test to evaluate severity of disease in patients with chronic hepatitis C virus infection. Aliment. Pharmacol. Ther..

[171] Lauterburg B.H., Grattagliano I., Gmur R., Stalder M., Hildebrand P. (1995). Noninvasive assessment of the effect of xenobiotics on mitochondrial function in human beings: Studies with acetylsalicylic acid and ethanol with the use of the carbon 13-labeled ketoisocaproate breath test. J. Lab. Clin. Med..

[172] Molina-Molina E., Shanmugam H., Di Palo D., Grattagliano I., Portincasa P. (2021). Exploring Liver Mitochondrial Function by (13)C-Stable Isotope Breath Tests: Implications in Clinical Biochemistry. Methods Mol. Biol..

[173] Lauterburg B.H., Liang D., Schwarzenbach F.A., Breen K.J. (1993). Mitochondrial dysfunction in alcoholic patients as assessed by breath analysis. Hepatology.

[174] Armuzzi A., Marcoccia S., Zocco M.A., De Lorenzo A., Grieco A., Tondi P., Pola P., Gasbarrini G., Gasbarrini A. (2000). Non-Invasive assessment of human hepatic mitochondrial function through the 13C-methionine breath test. Scand. J. Gastroenterol..

[175] Russmann S., Junker E., Lauterburg B.H. (2002). Remethylation and transsulfuration of methionine in cirrhosis: Studies with L-[H3-methyl-1-C]methionine. Hepatology.

[176] Banasch M., Ellrichmann M., Tannapfel A., Schmidt W.E., Goetze O. (2011). The non-invasive (13)C-methionine breath test detects hepatic mitochondrial dysfunction as a marker of disease activity in non-alcoholic steatohepatitis. Eur. J. Med. Res..

[177] Banasch M., Emminghaus R., Ellrichmann M., Schmidt W.E., Goetze O. (2008). Longitudinal effects of hepatitis C virus treatment on hepatic mitochondrial dysfunction assessed by C-methionine breath test. Aliment. Pharmacol. Ther..

[178] Spahr L., Negro F., Leandro G., Marinescu O., Goodman K.J., Rubbia-Brandt L., Jordan M., Hadengue A. (2003). Impaired hepatic mitochondrial oxidation using the 13C-methionine breath test in patients with macrovesicular steatosis and patients with cirrhosis. Med. Sci. Monit. Int. Med. J. Exp. Clin. Res..

[179] Stuwe S.H., Goetze O., Arning L., Banasch M., Schmidt W.E., Schols L., Saft C. (2011). Hepatic mitochondrial dysfunction in Friedreich ataxia. BMC Neurol..

[180] Miele L., Grieco A., Armuzzi A., Candelli M., Forgione A., Gasbarrini A., Gasbarrini G. (2003). Hepatic mitochondrial beta-oxidation in patients with nonalcoholic steatohepatitis assessed by 13 C-octanoate breath test. Am. J. Gastroenterol..

[181] Schneider A.R.J., Kraut C., Lindenthal B., Braden B., Caspary W.F., Stein J. (2005). Total body metabolism of 13C-octanoic acid is preserved in patients with non-alcoholic steatohepatitis, but differs between women and men. Eur. J. Gastroenterol. Hepatol..

[182] Grattagliano I., Vendemiale G., Lauterburg B.H. (1999). Reperfusion injury of the liver: Role of mitochondria and protection by glutathione ester. J. Surg. Res..

[183] Berthold H.K., Giesen T.A., Gouni-Berthold I. (2009). The stable isotope ketoisocaproic acid breath test as a measure of hepatic decarboxylation capacity: A quantitative analysis in normal subjects after oral and intravenous administration. Liver Int..

[184] Witschi A., Mossi S., Meyer B., Junker E., Lauterburg B.H. (1994). Mitochondrial function reflected by the decarboxylation of [13C]ketoisocaproate is impaired in alcoholics. Alcohol. Clin. Exp. Res..

[185] Bendtsen P., Hannestad U., Pahlsson P. (1998). Evaluation of the carbon 13-labeled Ketoisocaproate breath test to assess mitochondrial dysfunction in patients with high alcohol consumption. Alcohol. Clin. Exp. Res..

[186] Pugh R.N., Murray-Lyon I.M., Dawson J.L., Pietroni M.C., Williams R. (1973). Transection of the oesophagus for bleeding oesophageal varices. Br. J. Surg..

[187] Pessayre D., Mansouri A., Haouzi D., Fromenty B. (1999). Hepatotoxicity due to mitochondrial dysfunction. Cell Biol. Toxicol..

[188] Kass G.E., Price S.C. (2008). Role of mitochondria in drug-induced cholestatic injury. Clin. Liver Dis..

[189] Milazzo L., Piazza M., Sangaletti O., Gatti N., Cappelletti A., Adorni F., Antinori S., Galli M., Moroni M., Riva A. (2005). [13C]Methionine breath test: A novel method to detect antiretroviral drug-related mitochondrial toxicity. J. Antimicrob. Chemother..

[190] Li Y., Boehning D.F., Qian T., Popov V.L., Weinman S.A. (2007). Hepatitis C virus core protein increases mitochondrial ROS production by stimulation of Ca2+ uniporter activity. FASEB J..

[191] Jaeschke H., McGill M.R., Ramachandran A. (2012). Oxidant stress, mitochondria, and cell death mechanisms in drug-induced liver injury: Lessons learned from acetaminophen hepatotoxicity. Drug Metab. Rev..

[192] Nakagawa Y., Suzuki T., Kamimura H., Nagai F. (2006). Role of mitochondrial membrane permeability transition in N-nitrosofenfluramine-induced cell injury in rat hepatocytes. Eur. J. Pharmacol..

[193] Trost L.C., Lemasters J.J. (1997). Role of the mitochondrial permeability transition in salicylate toxicity to cultured rat hepatocytes: Implications for the pathogenesis of Reye’s syndrome. Toxicol. Appl. Pharmacol..

[194] Mingatto F.E., dos Santos A.C., Rodrigues T., Pigoso A.A., Uyemura S.A., Curti C. (2000). Effects of nimesulide and its reduced metabolite on mitochondria. Br. J. Pharmacol..

[195] Danicke S., Diers S. (2013). Effects of ergot alkaloids on liver function of piglets as evaluated by the (13)C-methacetin and (13)C-alpha-ketoisocaproic acid breath test. Toxins.

[196] Storch K.J., Wagner D.A., Burke J.F., Young V.R. (1988). Quantitative study in vivo of methionine cycle in humans using [methyl-2H3]- and [1-13C]methionine. Am. J. Physiol..

[197] Candelli M., Miele L., Armuzzi A., Nista E.C., Pignataro G., Fini L., Cazzato I.A., Zocco M.A., Bartolozzi F., Gasbarrini G. (2008). 13C-methionine breath tests for mitochondrial liver function assessment. Eur. Rev. Med Pharmacol. Sci..

[198] Duro D., Duggan C., Valim C., Bechard L., Fitzgibbons S., Jaksic T., Yu Y.M. (2009). Novel intravenous (13)C-methionine breath test as a measure of liver function in children with short bowel syndrome. J. Pediatric Surg..

[199] Durr A., Cossee M., Agid Y., Campuzano V., Mignard C., Penet C., Mandel J.L., Brice A., Koenig M. (1996). Clinical and genetic abnormalities in patients with Friedreich’s ataxia. N. Engl. J. Med..

[200] Walton M.E., Ebert D., Haller R.G. (2003). Octanoate oxidation measured by 13C-NMR spectroscopy in rat skeletal muscle, heart, and liver. J. Appl. Physiol..

[201] Shalev T., Aeed H., Sorin V., Shahmurov M., Didkovsky E., Ilan Y., Avni Y., Shirin H. (2010). Evaluation of the 13C-octanoate breath test as a surrogate marker of liver damage in animal models. Dig. Dis. Sci..

[202] Van de Casteele M., Luypaerts A., Geypens B., Fevery J., Ghoos Y., Nevens F. (2003). Oxidative breakdown of octanoic acid is maintained in patients with cirrhosis despite advanced disease. Neurogastroenterol. Motil..

[203] Ghoos Y.F., Maes B.D., Geypens B.J., Mys G., Hiele M.I., Rutgeerts P.J., Vantrappen G. (1993). Measurement of gastric emptying rate of solids by means of a carbon-labeled octanoic acid breath test. Gastroenterology.

[204] Promrat K., Kleiner D.E., Niemeier H.M., Jackvony E., Kearns M., Wands J.R., Fava J.L., Wing R.R. (2010). Randomized controlled trial testing the effects of weight loss on nonalcoholic steatohepatitis. Hepatology.

[205] Keating S.E., Hackett D.A., George J., Johnson N.A. (2012). Exercise and non-alcoholic fatty liver disease: A systematic review and meta-analysis. J. Hepatol..

[206] Keating S.E., Hackett D.A., Parker H.M., O’Connor H.T., Gerofi J.A., Sainsbury A., Baker M.K., Chuter V.H., Caterson I.D., George J. (2015). Effect of aerobic exercise training dose on liver fat and visceral adiposity. J. Hepatol..

[207] Vilar-Gomez E., Martinez-Perez Y., Calzadilla-Bertot L., Torres-Gonzalez A., Gra-Oramas B., Gonzalez-Fabian L., Friedman S.L., Diago M., Romero-Gomez M. (2015). Weight Loss Through Lifestyle Modification Significantly Reduces Features of Nonalcoholic Steatohepatitis. Gastroenterology.

[208] Petersen K.F., Dufour S., Befroy D., Lehrke M., Hendler R.E., Shulman G.I. (2005). Reversal of nonalcoholic hepatic steatosis, hepatic insulin resistance, and hyperglycemia by moderate weight reduction in patients with type 2 diabetes. Diabetes.

[209] Musso G., Cassader M., Rosina F., Gambino R. (2012). Impact of current treatments on liver disease, glucose metabolism and cardiovascular risk in non-alcoholic fatty liver disease (NAFLD): A systematic review and meta-analysis of randomised trials. Diabetologia.

[210] Cerqueira F.M., Cunha F.M.d., Silva C.C., Chausse B., Romano R.L., Garcia C., Colepicolo P., Medeiros M.H.G.d., Kowaltowski A.J. (2011). Redox state, insulin sensitivity and aging. Resumos.

[211] Kowaltowski A.J. (2011). Caloric restriction and redox state: Does this diet increase or decrease oxidant production?. Redox Rep..

[212] Walsh M.E., Shi Y., Van Remmen H. (2014). The effects of dietary restriction on oxidative stress in rodents. Free Radic. Biol. Med..

[213] Bower G., Toma T., Harling L., Jiao L.R., Efthimiou E., Darzi A., Athanasiou T., Ashrafian H. (2015). Bariatric Surgery and Non-Alcoholic Fatty Liver Disease: A Systematic Review of Liver Biochemistry and Histology. Obes. Surg..

[214] Mathurin P., Hollebecque A., Arnalsteen L., Buob D., Leteurtre E., Caiazzo R., Pigeyre M., Verkindt H., Dharancy S., Louvet A. (2009). Prospective study of the long-term effects of bariatric surgery on liver injury in patients without advanced disease. Gastroenterology.

[215] Ekstedt M., Franzen L.E., Holmqvist M., Bendtsen P., Mathiesen U.L., Bodemar G., Kechagias S. (2009). Alcohol consumption is associated with progression of hepatic fibrosis in non-alcoholic fatty liver disease. Scand. J. Gastroenterol..

[216] Pessayre D., Berson A., Fromenty B., Mansouri A. (2001). Mitochondria in steatohepatitis. Semin. Liver Dis..

[217] Thoma C., Day C.P., Trenell M.I. (2012). Lifestyle interventions for the treatment of non-alcoholic fatty liver disease in adults: A systematic review. J. Hepatol..

[218] Li Z., Li Y., Zhang H.X., Guo J.R., Lam C.W.K., Wang C.Y., Zhang W. (2019). Mitochondria-Mediated Pathogenesis and Therapeutics for Non-Alcoholic Fatty Liver Disease. Mol. Nutr. Food Res..

[219] Stevanovic J., Beleza J., Coxito P., Ascensao A., Magalhaes J. (2020). Physical exercise and liver “fitness”: Role of mitochondrial function and epigenetics-related mechanisms in non-alcoholic fatty liver disease. Mol. Metab..

[220] Venditti P., Di Meo S. (1996). Antioxidants, tissue damage, and endurance in trained and untrained young male rats. Arch. Biochem. Biophys..

[221] Ascensao A., Martins M.J., Santos-Alves E., Goncalves I.O., Portincasa P., Oliveira P.J., Magalhaes J. (2013). Modulation of hepatic redox status and mitochondrial metabolism by exercise: Therapeutic strategy for liver diseases. Mitochondrion.

[222] Samuel V.T., Shulman G.I. (2012). Mechanisms for insulin resistance: Common threads and missing links. Cell.

[223] Neuschwander-Tetri B.A., Loomba R., Sanyal A.J., Lavine J.E., Van Natta M.L., Abdelmalek M.F., Chalasani N., Dasarathy S., Diehl A.M., Hameed B. (2015). Farnesoid X nuclear receptor ligand obeticholic acid for non-cirrhotic, non-alcoholic steatohepatitis (FLINT): A multicentre, randomised, placebo-controlled trial. Lancet.

[224] Mudaliar S., Henry R.R., Sanyal A.J., Morrow L., Marschall H.U., Kipnes M., Adorini L., Sciacca C.I., Clopton P., Castelloe E. (2013). Efficacy and safety of the farnesoid X receptor agonist obeticholic acid in patients with type 2 diabetes and nonalcoholic fatty liver disease. Gastroenterology.

[225] Ratziu V., Sanyal A.J., Loomba R., Rinella M., Harrison S., Anstee Q.M., Goodman Z., Bedossa P., MacConell L., Shringarpure R. (2019). REGENERATE: Design of a pivotal, randomised, phase 3 study evaluating the safety and efficacy of obeticholic acid in patients with fibrosis due to nonalcoholic steatohepatitis. Contemp. Clin. Trials.

[226] Chen Y.S., Liu H.M., Lee T.Y. (2019). Ursodeoxycholic Acid Regulates Hepatic Energy Homeostasis and White Adipose Tissue Macrophages Polarization in Leptin-Deficiency Obese Mice. Cells.

[227] Xie C., Jiang C., Shi J., Gao X., Sun D., Sun L., Wang T., Takahashi S., Anitha M., Krausz K.W. (2017). An Intestinal Farnesoid X Receptor-Ceramide Signaling Axis Modulates Hepatic Gluconeogenesis in Mice. Diabetes.

[228] Ferramosca A., Di Giacomo M., Zara V. (2017). Antioxidant dietary approach in treatment of fatty liver: New insights and updates. World J. Gastroenterol. WJG.

[229] Ding S., Jiang J., Zhang G., Bu Y., Zhang G., Zhao X. (2017). Resveratrol and caloric restriction prevent hepatic steatosis by regulating SIRT1-autophagy pathway and alleviating endoplasmic reticulum stress in high-fat diet-fed rats. PLoS ONE.

[230] Tian Y., Ma J., Wang W., Zhang L., Xu J., Wang K., Li D. (2016). Resveratrol supplement inhibited the NF-κB inflammation pathway through activating AMPKα-SIRT1 pathway in mice with fatty liver. Mol. Cell. Biochem..

[231] Shang J., Chen L.L., Xiao F.X., Sun H., Ding H.C., Xiao H. (2008). Resveratrol improves non-alcoholic fatty liver disease by activating AMP-activated protein kinase. Acta Pharmacol. Sin..

[232] Asin-Cayuela J., Manas A.R., James A.M., Smith R.A., Murphy M.P. (2004). Fine-tuning the hydrophobicity of a mitochondria-targeted antioxidant. FEBS Lett..

[233] Rokitskaya T.I., Klishin S.S., Severina I.I., Skulachev V.P., Antonenko Y.N. (2008). Kinetic analysis of permeation of mitochondria-targeted antioxidants across bilayer lipid membranes. J. Membr. Biol..

[234] Smith R.A., Porteous C.M., Gane A.M., Murphy M.P. (2003). Delivery of bioactive molecules to mitochondria in vivo. Proc. Natl. Acad. Sci. USA.

[235] Grattagliano I., Diogo C.V., Mastrodonato M., de Bari O., Persichella M., Wang D.Q., Liquori A., Ferri D., Carratu M.R., Oliveira P.J. (2013). A silybin-phospholipids complex counteracts rat fatty liver degeneration and mitochondrial oxidative changes. World J. Gastroenterol. WJG.

[236] Vecchione G., Grasselli E., Cioffi F., Baldini F., Oliveira P.J., Sardao V.A., Cortese K., Lanni A., Voci A., Portincasa P. (2017). The Nutraceutic Silybin Counteracts Excess Lipid Accumulation and Ongoing Oxidative Stress in an In Vitro Model of Non-Alcoholic Fatty Liver Disease Progression. Front. Nutr..

[237] Wu N., Zu Y., Fu Y., Kong Y., Zhao J., Li X., Li J., Wink M., Efferth T. (2010). Antioxidant activities and xanthine oxidase inhibitory effects of extracts and main polyphenolic compounds obtained from *Geranium sibiricum* L.. J. Agric. Food Chem..

[238] Ling W.H., Shen T.R., Tang X.L., Jiang X.W. (2016). Anthocyanins Improved Mitochondrial Dysfunction in Mice of Non-alcoholic Fatty Liver Disease Induced by High Fat Diet. Faseb J..

[239] Tang X., Shen T., Jiang X., Xia M., Sun X., Guo H., Ling W. (2015). Purified anthocyanins from bilberry and black currant attenuate hepatic mitochondrial dysfunction and steatohepatitis in mice with methionine and choline deficiency. J. Agric. Food Chem..

[240] Zeng X., Yang J., Hu O., Huang J., Ran L., Chen M., Zhang Y., Zhou X., Zhu J., Zhang Q. (2019). Dihydromyricetin ameliorates nonalcoholic fatty liver disease by improving mitochondrial respiratory capacity and redox homeostasis through modulation of SIRT3 signaling. Antioxid. Redox Signal..

[241] Teodoro J.S., Duarte F.V., Gomes A.P., Varela A.T., Peixoto F.M., Rolo A.P., Palmeira C.M. (2013). Berberine reverts hepatic mitochondrial dysfunction in high-fat fed rats: A possible role for SirT3 activation. Mitochondrion.

[242] Echeverria F., Valenzuela R., Bustamante A., Alvarez D., Ortiz M., Espinosa A., Illesca P., Gonzalez-Manan D., Videla L.A. (2019). High-fat diet induces mouse liver steatosis with a concomitant decline in energy metabolism: Attenuation by eicosapentaenoic acid (EPA) or hydroxytyrosol (HT) supplementation and the additive effects upon EPA and HT co-administration. Food Funct..

[243] Schwimmer J.B., Lavine J.E., Wilson L.A., Neuschwander-Tetri B.A., Xanthakos S.A., Kohli R., Barlow S.E., Vos M.B., Karpen S.J., Molleston J.P. (2016). In children with nonalcoholic fatty liver disease, cysteamine bitartrate delayed release improves liver enzymes but does not reduce disease activity scores. Gastroenterology.

[244] Dohil R., Schmeltzer S., Cabrera B.L., Wang T., Durelle J., Duke K.B., Schwimmer J.B., Lavine J.E. (2011). Enteric-coated cysteamine for the treatment of paediatric non-alcoholic fatty liver disease. Aliment. Pharmacol. Ther..

[245] Ye J.H., Chao J., Chang M.L., Peng W.H., Cheng H.Y., Liao J.W., Pao L.H. (2016). Pentoxifylline ameliorates non-alcoholic fatty liver disease in hyperglycaemic and dyslipidaemic mice by upregulating fatty acid beta-oxidation. Sci. Rep..

[246] Zein C.O., Lopez R., Yerian L., Anderson K.A., McCullough A.J., Rinella M.E. (2012). 932 Pentoxifylline Improves Non-Invasive Serum Markers of Fibrosis: Combined Results From 2 Randomized, Placebo-Controlled Trials. Gastroenterology.

[247] Zein C.O., Yerian L.M., Gogate P., Lopez R., Kirwan J.P., Feldstein A.E., McCullough A.J. (2011). Pentoxifylline improves nonalcoholic steatohepatitis: A randomized placebo-controlled trial. Hepatology.

[248] Rendon D.A. (2015). Letter to the Editor: The bioenergetics of hepatic mitochondria isolated from avocado oil-treated rats: Typical experimental errors in the study of the bioenergetics of isolated mitochondria. J. Bioenerg. Biomembr..

[249] Ortiz-Avila O., Gallegos-Corona M.A., Sanchez-Briones L.A., Calderon-Cortes E., Montoya-Perez R., Rodriguez-Orozco A.R., Campos-Garcia J., Saavedra-Molina A., Mejia-Zepeda R., Cortes-Rojo C. (2015). Protective effects of dietary avocado oil on impaired electron transport chain function and exacerbated oxidative stress in liver mitochondria from diabetic rats. J. Bioenerg. Biomembr..

[250] Garcia-Berumen C.I., Olmos-Orizaba B.E., Marquez-Ramirez C.A., Orozco A.R.R., Gonzalez-Cortez A., Saavedra-Molina A., Montoya-Perez R., Cortes-Rojo C. (2019). Avocado Oil Ameliorates Non-Alcoholic Fatty Liver Disease by Down-Regulating Inflammatory Cytokines and Improving Mitochondrial Dynamics. FASEB J..

[251] Sanyal A., Charles E.D., Neuschwander-Tetri B.A., Loomba R., Harrison S.A., Abdelmalek M.F., Lawitz E.J., Halegoua-DeMarzio D., Kundu S., Noviello S. (2019). Pegbelfermin (BMS-986036), a PEGylated fibroblast growth factor 21 analogue, in patients with non-alcoholic steatohepatitis: A randomised, double-blind, placebo-controlled, phase 2a trial. Lancet.

[252] Staels B., Rubenstrunk A., Noel B., Rigou G., Delataille P., Millatt L.J., Baron M., Lucas A., Tailleux A., Hum D.W. (2013). Hepatoprotective effects of the dual peroxisome proliferator-activated receptor alpha/delta agonist, GFT505, in rodent models of nonalcoholic fatty liver disease/nonalcoholic steatohepatitis. Hepatology.

[253] Ratziu V., Harrison S.A., Francque S., Bedossa P., Lehert P., Serfaty L., Romero-Gomez M., Boursier J., Abdelmalek M., Caldwell S. (2016). Elafibranor, an Agonist of the Peroxisome Proliferator-Activated Receptor-alpha and -delta, Induces Resolution of Nonalcoholic Steatohepatitis Without Fibrosis Worsening. Gastroenterology.

[254] Tong W., Ju L., Qiu M., Xie Q., Chen Y., Shen W., Sun W., Wang W., Tian J. (2016). Liraglutide ameliorates non-alcoholic fatty liver disease by enhancing mitochondrial architecture and promoting autophagy through the SIRT1/SIRT3-FOXO3a pathway. Hepatol. Res. Off. J. Jpn. Soc. Hepatol..

[255] He L., Sabet A., Djedjos S., Miller R., Sun X., Hussain M.A., Radovick S., Wondisford F.E. (2009). Metformin and insulin suppress hepatic gluconeogenesis through phosphorylation of CREB binding protein. Cell.

[256] Sun L., Yuan Q., Xu T., Yao L., Feng J., Ma J., Wang L., Lu C., Wang D. (2017). Pioglitazone Improves Mitochondrial Function in the Remnant Kidney and Protects against Renal Fibrosis in 5/6 Nephrectomized Rats. Front. Pharmacol..

[257] Harrison S.A., Alkhouri N., Davison B.A., Sanyal A., Edwards C., Colca J.R., Lee B.H., Loomba R., Cusi K., Kolterman O. (2020). Insulin sensitizer MSDC-0602K in non-alcoholic steatohepatitis: A randomized, double-blind, placebo-controlled phase IIb study. J. Hepatol..

[258] Iruarrizaga-Lejarreta M., Varela-Rey M., Fernandez-Ramos D., Martinez-Arranz I., Delgado T.C., Simon J., Juan V.G., delaCruz-Villar L., Azkargorta M., Lavin J.L. (2017). Role of Aramchol in steatohepatitis and fibrosis in mice. Hepatol. Commun..

[259] Safadi R., Konikoff F.M., Mahamid M., Zelber-Sagi S., Halpern M., Gilat T., Oren R., Group F. (2014). The fatty acid-bile acid conjugate Aramchol reduces liver fat content in patients with nonalcoholic fatty liver disease. Clin. Gastroenterol. Hepatol..

[260] Dai J., Liang K., Zhao S., Jia W., Liu Y., Wu H., Lv J., Cao C., Chen T., Zhuang S. (2018). Chemoproteomics reveals baicalin activates hepatic CPT1 to ameliorate diet-induced obesity and hepatic steatosis. Proc. Natl. Acad. Sci. USA.

[261] Fazzari M., Chartoumpekis D., Li L., Guimaraes D.A., Shiva S., Freeman B.A., Khoo N. (2017). Nitro-oleic Acid Protects Mice from Diet-Induced Hepatic Steatosis and Insulin Resistance without the Adverse Side Effects of Thiazolidinediones. Free Radic. Biol. Med..

[262] Cho J., Zhang Y., Park S.-Y., Joseph A.-M., Han C., Park H.-J., Kalavalapalli S., Chun S.-K., Morgan D., Kim J.-S. (2017). Mitochondrial ATP transporter depletion protects mice against liver steatosis and insulin resistance. Nat. Commun..

[263] Amanat S., Eftekhari M.H., Fararouei M., Bagheri Lankarani K., Massoumi S.J. (2018). Genistein supplementation improves insulin resistance and inflammatory state in non-alcoholic fatty liver patients: A randomized, controlled trial. Clin. Nutr..

[264] Lawitz E.J., Neff G., Ruane P.J., Younes Z., Zhang J., Jia C., Chuang J., Huss R., Chung C., Subramanian M. (2019). Fenofibrate mitigates increases in serum triglycerides due to the ACC inhibitor firsocostat in patients with advanced fibrosis due to NASH: A phase 2 randomized trial. Hepatology.

[265] Fu A., Shi X., Zhang H., Fu B. (2017). Mitotherapy for Fatty Liver by Intravenous Administration of Exogenous Mitochondria in Male Mice. Front. Pharmacol..

[266] Ajith T.A. (2018). Role of mitochondria and mitochondria-targeted agents in non-alcoholic fatty liver disease. Clin. Exp. Pharmacol. Physiol..

[267] Pessayre D., Fromenty B. (2005). NASH: A mitochondrial disease. J. Hepatol..

[268] Winchell H.S., Wiley K. (1970). Considerations in analysis of breath 14CO2 data. J. Nucl. Med. Off. Publ. Soc. Nucl. Med..

